# Evaluation of the offline-coupled GFSv15–FV3–CMAQv5.0.2 in support of the next-generation National Air Quality Forecast Capability over the contiguous United States

**DOI:** 10.5194/gmd-14-3969-2021

**Published:** 2021-06-29

**Authors:** Xiaoyang Chen, Yang Zhang, Kai Wang, Daniel Tong, Pius Lee, Youhua Tang, Jianping Huang, Patrick C. Campbell, Jeff Mcqueen, Havala O. T. Pye, Benjamin N. Murphy, Daiwen Kang

**Affiliations:** 1 Department of Civil and Environmental Engineering, Northeastern University, Boston, MA 02115, USA; 2 Department of Atmospheric, Oceanic and Earth Sciences, George Mason University, Fairfax, VA 22030, USA; 3 Center for Spatial Information Science and System, George Mason University, Fairfax, VA 22030, USA; 4 Air Resources Laboratory, National Oceanic and Atmospheric Administration, College Park, MD 20740, USA; 5 National Oceanic and Atmospheric Administration/National Centers for Environmental Prediction/Environmental Modeling Center, College Park, MD 20740, USA; 6 IM Systems Group, Rockville, MD 20852, USA; 7 Office of Research and Development, U.S. Environmental Protection Agency, Research Triangle Park, NC 27711, USA

## Abstract

As a candidate for the next-generation National Air Quality Forecast Capability (NAQFC), the meteorological forecast from the Global Forecast System with the new Finite Volume Cube-Sphere dynamical core (GFS–FV3) will be applied to drive the chemical evolution of gases and particles described by the Community Multiscale Air Quality modeling system. CMAQv5.0.2, a historical version of CMAQ, has been coupled with the North American Mesoscale Forecast System (NAM) model in the current operational NAQFC. An experimental version of the NAQFC based on the offline-coupled GFS–FV3 version 15 with CMAQv5.0.2 modeling system (GFSv15–CMAQv5.0.2) has been developed by the National Oceanic and Atmospheric Administration (NOAA) to provide real-time air quality forecasts over the contiguous United States (CONUS) since 2018. In this work, comprehensive region-specific, time-specific, and categorical evaluations are conducted for meteorological and chemical forecasts from the offline-coupled GFSv15–CMAQv5.0.2 for the year 2019. The forecast system shows good overall performance in forecasting meteorological variables with the annual mean biases of −0.2 °C for temperature at 2 m, 0.4% for relative humidity at 2 m, and 0.4 m s^−1^ for wind speed at 10 m compared to the METeorological Aerodrome Reports (METAR) dataset. Larger biases occur in seasonal and monthly mean forecasts, particularly in spring. Although the monthly accumulated precipitation forecasts show generally consistent spatial distributions with those from the remote-sensing and ensemble datasets, moderate-to-large biases exist in hourly precipitation forecasts compared to the Clean Air Status and Trends Network (CASTNET) and METAR. While the forecast system performs well in forecasting ozone (O_3_) throughout the year and fine particles with a diameter of 2.5 μm or less (PM_2.5_) for warm months (May–September), it significantly overpredicts annual mean concentrations of PM_2.5_. This is due mainly to the high predicted concentrations of fine fugitive and coarse-mode particle components. Underpredictions in the southeastern US and California during summer are attributed to missing sources and mechanisms of secondary organic aerosol formation from biogenic volatile organic compounds (VOCs) and semivolatile or intermediate-volatility organic compounds. This work demonstrates the ability of FV3-based GFS in driving the air quality forecasting. It identifies possible underlying causes for systematic region- and time-specific model biases, which will provide a scientific basis for further development of the next-generation NAQFC.

## 1 Introduction

Three-dimensional air quality models (3-D AQMs) have been widely applied in real-time air quality forecasting (RT-AQF) since the 1990s in the US (Stein et al., 2000; McHenry et al., 2004; Zhang et al., 2012a). The developments and applications of the national air quality forecasting systems based on 3-D AQMs were conducted in the 2000s (Kang et al., 2005; Otte et al., 2005; McKeen et al., 2005, 2007, 2009). Since then, improvements and significant progress have been achieved in RT-AQF through the further development of AQMs and the use of advanced techniques. For example, more air pollutants in the products, more detailed gas-phase chemical mechanisms and aerosol chemistry, and the implementation of chemical data assimilation were available (Zhang et al., 2012b; Lee et al., 2017). Various AQMs, coupled with meteorological models in either an online or offline manner, were developed and applied in RT-AQF (e.g., Chuang et al., 2011; Lee et al., 2011; Žabkar et al., 2015; Ryan, 2016). The early version of the National Air Quality Forecast Capability (NAQFC) was jointly developed by the US National Oceanic and Atmospheric Administration (NOAA) and the U.S. Environmental Protection Agency (EPA) to provide forecasts of ozone (O_3_) over the northeastern US (Eder et al., 2006). Since the first operational version over the contiguous United States (CONUS) (Eder et al., 2009), the NAQFC has been continuously updated and developed to provide more forecasting products (including O_3_, smoke, dust, and particulate matter with a diameter of 2.5 μm or less (PM_2.5_)) with increasing accuracy (Mathur et al., 2008; Stajner et al., 2011; Lee et al., 2017).

The forecast skill of a historical NAQFC, which was based on the North American Mesoscale Forecast System (NAM) model (Black, 1994) and the Community Multiscale Air Quality Modeling System version 4.6 (CMAQv4.6), over CONUS during the year 2008 was evaluated by Kang et al. (2010a) for operational O_3_ and experimental PM_2.5_ products. Overall, maximum 8 h O_3_ was slightly overpredicted over the CONUS during the summer, with a mean bias (MB), normalized mean bias (NMB), and correlation coefficient (Corr) of 3.2 ppb, 6.8%, and 0.65, respectively. The performance of predicted daily mean PM_2.5_ varied: there was an underprediction during the warm season and an overprediction in the cool season. The MBs and NMBs during warm/cool seasons were −2.3/4.5 μg m^−3^ and −19.6%/45.1%, respectively. The current version of the US NOAA’s operational NAQFC has provided the air quality forecast to the public for O_3_ and PM_2.5_ at a horizontal grid resolution of 12 km over CONUS since 2015. It is currently based on the CMAQv5.0.2 (released May 2014) (U.S. EPA, 2014) coupled offline with the NAM model. Daily mean PM_2.5_ was underpredicted during warm months (May and July 2014) and overpredicted during a cool month (January 2015) over CONUS (Lee et al., 2017).

Efforts have been made to reduce the seasonal and region-specific biases in the historical and current NAQFC. Development and implementation of an analog ensemble bias correction approach was applied to the operational NAQFC to improve forecast performance in PM_2.5_ predictions (Huang et al., 2017). Kang et al. (2008, 2010b) investigated the Kalman filter (KF) bias-adjustment technique for operational use in the NAQFC system. The KF bias-adjusted forecasts showed significant improvement in both O_3_ and PM_2.5_ for discrete and categorical evaluations. However, limitations in the underlying models and the bias correction or adjustment approaches need further improvement. Characterizing the current NAQFC forecasting skill and identifying the underlying causes for region- and time-specific biases can result in further development of the NAQFC system and improved pollutant predictions.

As the NOAA Environmental Modeling Center (EMC) has transitioned to devote its full resources to the development of an ensemble model based on the Finite Volume Cube-Sphere Dynamical Core (FV3), NAM has been no longer updated since March 2017. The FV3 dynamic core will eventually replace all current NOAA National Centers for Environmental Prediction (NCEP) mesoscale models used for forecasting. The FV3 dynamical core was implemented in the operational Global Forecast System as version 15 (GFSv15) in July 2019.

The NOAA National Weather Service (NWS) is currently coordinating an effort to inline a regional-scale meteorological model based on the same FV3 dynamic core as that in GFSv15 to be coupled with an atmospheric chemistry model partially based on CMAQ. The inline system is expected to be the next generation of NAQFC and to be implemented a few years into the future. An interim system, offline coupling the recent CMAQ with FV3-based GFS is regarded as a candidate NAQFC to replace the current NAM–CMAQ system before the inline system is applied in operational air quality forecasting. To support this new development of the interim NAQFC, a prototype of the offline-coupled GFSv15 with CMAQv5.0.2 (GFSv15–CMAQv5.0.2) has been developed and applied by the NOAA for RT-AQF over CONUS since 2018 (Huang et al., 2018, 2019). In this work, the meteorological and air quality forecasts from the offline-coupled GFSv15–CMAQv5.0.2 system are comprehensively evaluated for the year of 2019. The main objectives of this work are to (1) evaluate the forecast skills of the experimental prototype of the GFSv15–CMAQv5.0.2 system, (2) identify the major model biases, in particular, systematic biases and persistent region- and time-specific biases in major species, and (3) investigate underlying causes for the biases to provide a scientific basis for improving the model representations of chemical processes and developing science-based bias correction methods for O_3_ and PM_2.5_ forecasts. This work will support NAQFC’s further development and improvement through enhancing its forecasting abilities and generating a benchmark for the interim NAQFC that is being developed by NOAA based on the offline-coupled GFS–FV3 v16 with CMAQv5.3 (NACC–CMAQ) (Campbell et al., 2020). Eventually, the latest version of CMAQ (version 5.3), which has updates in gas-phase chemistry (Yarwood et al., 2010; Emery et al., 2015; Luecken et al., 2019), lightning nitric oxide (LNO) production schemes (Kang et al., 2019a, b), and secondary aerosol formation (in particular, secondary organic aerosol) (e.g., Pye et al., 2013, 2017; Murphy et al., 2017) among other things, will be coupled with GFS–FV3 v16 and be implemented in the interim operational NAQFC.

## 2 Model system and evaluation protocols

### 2.1 Description and configuration of offline-coupled GFSv15–CMAQv5.0.2

FV3 is a dynamical core for atmospheric numerical models developed by the Geophysical Fluid Dynamics Laboratory (GFDL) (Putman and Lin, 2007). It is a modern and extended version of the original FV core with a cubed-sphere grid design and more computationally efficient solvers. It was selected for implementation into the GFS as the next generation dynamical core in 2016 (C. Zhang et al., 2019). The GFS–FV3 v15 (GFSv15) has been operational since June 2019. The GFSv15 uses the Rapid Radiative Transfer Method for General Circulation Models (RRTMG) scheme for shortwave or longwave radiation (Mlawer et al., 1997; Iacono et al., 2000; Clough et al., 2005), the Hybrid eddy-diffusivity mass-flux (EDMF) scheme for the planetary boundary layer (PBL) (National Centers for Environmental Prediction, 2019a), the Noah Land Surface Model (LSM) scheme for the land surface option (Chen et al., 1997), the simplified Arakawa–Schubert (SAS) deep convection for cumulus parameterization (Arakawa and Schubert, 1974; Grell, 1993), and a more advanced GFDL microphysics scheme for microphysics (National Centers for Environmental Prediction, 2019b). An interface preprocessor has been developed by NOAA to interpolate data, transfer coordinates, and convert the GFSv15 outputs into the data format required by CMAQv5.0.2 (Huang et al., 2018, 2019). The original outputs from GFSv15, which have a horizontal grid with 13 km resolution and a Lagrangian vertical coordinate with 64 layers in I/O format for the NCEP models using the NOAA Environmental Modeling System (NEMSIO), are processed to Lambert conformal conic projection by PREMAQ, a preprocessor, to recast the meteorological fields for CMAQ into an Arakawa C-staggering grid (Arakawa and Lamb, 1977) with a 12 km horizontal resolution and 35 vertical layers (Table 1). The first 72 h in 12:00 UTC forecast cycles from GFSv15 are used to drive the air quality forecast by the offline-coupled GFSv15–CMAQv5.0.2 system.

CMAQ has been continuously developed by the U.S. EPA since the 1990s (Byun and Schere, 2006) and has been significantly updated in many atmospheric processes since then. Chemical boundary conditions for the GFSv15–CMAQv5.0.2 system are mainly from the global 3-D model of atmospheric chemistry driven by meteorological input from the Goddard Earth Observing System (GEOS-Chem). The lateral boundary condition for dust is from the outputs of the NOAA Environmental Modeling System GFS aerosol component (NGAC) (Lu et al., 2016). The anthropogenic emissions from area, mobile, and point sources in the National Emissions Inventory of the year 2014 version 2 (NEI 2014v2) are processed by the Sparse Matrix Operator Kernel Emissions (SMOKE) modeling system. The on-road mobile sources include all emissions from motor vehicles that operate on roadways, such as passenger cars, motorcycles, minivans, sport-utility vehicles, light-duty trucks, heavy-duty trucks, and buses. On-road mobile source emissions were processed using emission factors output from the Motor Vehicle Emissions Simulator (MOVES). SMOKE uses a combination of vehicle activity data, emission factors from MOVES, meteorology data, and temporal allocation information to estimate hourly, gridded on-road emissions. The non-road, agriculture, anthropogenic fugitive dust, non-elevated oil–gas, residential wood combustion, and other sectors are included in the area sources. The sectors of airports, commercial marine vessel (CMV), electric generating units (pt_egu), point sources related to oil and gas production (pt_oilgas), point sources that are not electric generating units (EGUs) nor related to oil and gas (pt_nonipm), and point sources outside the US (pt_other) are included in the point sources. The sulfur dioxide (SO_2_) and nitrogen oxide (NO*_x_*) from point sources in NEI 2005 are projected to the year 2019 following the methods used in Tang et al., (2015, 2017). The biomass burning emission inventory from the Blended Global Biomass Burning Emissions Product system (GBBEPx) (X. Zhang et al., 2019) is implemented for the forecast of forest fires. The GBBEPx fire emission is treated as one type of point source. Its heat flux is derived from satellite-retrieved fire radiative power (FRP) to drive fire plume rise. The GBBEPx is a near-real-time fire dataset. The fire emission implemented in the current forecast cycle comes from the historical fire observation, typically 1–2 d behind. In this system, we use land use information to classify fires as forest fire and other burning such as agriculture burning. We assume that only forest fire can last longer than 24 h. We assume that the forest fire emission will continue on day 2 and beyond. Other types of fires will be dropped. The plume rise of the point source will be driven by the meteorology and allocated to the 35 elevated layers in the GFSv15–CMAQv5.0.2 system by the PREMAQ preprocessing system. Biogenic emissions are calculated inline by the Biogenic Emission Inventory System (BEIS) version 3.14 (Schwede et al., 2005). Sea-salt emission is parameterized within CMAQv5.0.2. While the deposition velocities are calculated inline, the fertilizer ammonia bidirectional flux for inline emissions and deposition velocities is turned off. Detailed configurations of photolysis, gas-phase chemistry, aqueous chemistry, and aerosol chemistry for CMAQv5.0.2 are listed in Table 1.

### 2.2 Datasets and evaluation protocols

A comprehensive evaluation of the GFSv15–CMAQv5.0.2 forecasting system is conducted for both meteorological and chemical variables for the year 2019, including discrete, categorical, and region-specific evaluations. The products in the first 24 h of each 72 h forecast cycle are extracted and combined as a continuous, annual forecast. The evaluation of meteorological variables is carried out for those results from PREMAQ in the GFSv15–CMAQv5.0.2 system. Detailed information for datasets used in this study is listed in Table S1 in the Supplement. Observed hourly temperature at 2 m (*T*2), relative humidity at 2 m (RH2), precipitation (Precip), wind direction at 10 m (WD10), and wind speed at 10 m (WS10) are obtained from the Clean Air Status and Trends Network (CASTNET) and the METeorological Aerodrome Reports (METAR) datasets. The majority of CASTNET sites are suburban and rural sites. Approximately 1900 METAR sites over CONUS are used in this study (Fig. S1 in the Supplement). For the evaluation of precipitation, a threshold of ≥ 0.1 mm h^−1^ is used for valid records because CASTNET and METAR have different definitions of 0.0 mm h^−1^ values. In CASTNET, the records without any precipitation are given as 0.0 mm h^−1^, the same as those records with negligible precipitation. However, in METAR, the records without any precipitation are left blank, the same as an invalid record. The negligible precipitation is recorded as 0.0 mm h^−1^.

The air quality forecasting products that are evaluated include hourly O_3_, hourly PM_2.5_, maximum daily 8 h average O_3_ (MDA8 O_3_), and daily average PM_2.5_ (24 h average PM_2.5_) for chemical forecast. The AIRNow dataset is used for observed hourly O_3_ and PM_2.5_. We utilize the quality assurance/quality control (QA/QC) information from the AIRNow dataset to filter the invalid records. Remote-sensing data from the Global Precipitation Climatology Project (GPCP) and the Climatology-Calibrated Precipitation Analysis (CCPA) (Hou et al., 2014; Zhu and Luo, 2015) datasets are also used for the evaluation of precipitation. GPCP is a global precipitation dataset with a spatial resolution of 0.25° and a monthly temporal resolution. The CCPA uses linear regression and downscaling techniques to generate an analysis product of precipitation from two datasets: the NCEP Climate Prediction Center Unified Global Daily Gauge Analysis and the NCEP EMC Stage IV multi-sensor quantitative precipitation estimations (QPEs). The CCPA product with a spatial resolution in 0.125° and temporal resolution of an hour is used in this study. Satellite-based aerosol optical depth (AOD) at 550 nm from the Moderate Resolution Imaging Spectroradiometer (MODIS) Terra platform (Levy and Hsu, 2015) is used for the evaluation of monthly AOD. The statistical measures such as mean bias, the root mean square error (RMSE), the normalized mean bias, the normalized mean error (NME), and the correlation coefficient are used; more details about evaluation protocols are found in Zhang et al. (2009, 2016). The Taylor diagram (Taylor, 2001), which includes the correlations, NMBs, and the normalized standard deviations (NSDs), is used to present the overall performance (Wang et al., 2015). The NMBs ≤ 15% and NMEs ≤ 30% by Zhang et al. (2006) and NMBs (≤ 15% and ≤ 30%), NMEs (≤ 25% and ≤ 50%), and Corr (> 0.5 and > 0.4) for MDA8 O_3_ and 24 h PM_2.5_, respectively, by Emery et al. (2017) are regarded as performance criteria. Monthly, seasonal, and annual statistics and analysis are included. Seasonal analysis for O_3_ is separated into an O_3_ season (May–September) and a non-O_3_ season (January–April and October–December). Analysis for 10 CONUS regions, defined by the U.S. EPA (http://www.epa.gov/aboutepa, last access: 10 August 2020), is included and listed in Fig. S1c in the Supplement.

The metrics of false alarm ratio (FAR) and the hit rate (*H*) are used (Kang et al., 2005; Barnes et al., 2009) for categorical evaluation. Observed and forecasted MDA8 O_3_ and 24 h average PM_2.5_ values are divided into four classes based on whether the predicted and/or observed data fall above or below the air quality index (AQI) thresholds: (a) observed values ≤ thresholds and predicted values > thresholds, (b) observed and predicted values > thresholds, (c) observed and predicted values ≤ thresholds, and (d) observed values > thresholds and predicted values ≤ thresholds. The FAR and *H* are defined in Eqs. (1) and (2):

(1) $$\text{FAR}=\frac{a}{a+b}\times 100\%,$$

(2) $$H=\frac{b}{b+d}\times 100\%.$$

## 3 Evaluation of model forecast skills

### 3.1 Evaluation of meteorological forecasts

Discrete performance evaluation is conducted for postprocessed meteorological fields from the GFSv15–CMAQv5.0.2 system (Table 2). The GFSv15 can predict the boundary layer meteorological variables well. It has overall cold biases and wet biases for annual *T*2 and RH2 in 2019, respectively. It also overpredicts WS10, and underpredicts hourly precipitation. Despite the CASTNET siting being slightly different from that of METAR, the annual and most of the seasonal performance for the model shows a similar pattern in terms of bias for both the CASTNET and METAR networks. The mean biases of *T*2 are mostly within ±0.5°C except those in February and March compared to CASTNET (Table S2 in the Supplement). Underprediction is generally larger compared to CASTNET than METAR. For a spatial distribution of MB for seasonal *T*2 compared to METAR (Fig. S2 in the Supplement), cold biases are mainly found in the Midwest and western US where most of the CASTNET sites are located. GFSv15 usually underpredicts *T*2 on the west coast, the mountain states, and the Midwest. Overpredictions of *T*2 in the states of Kansas, Oklahoma, the areas near the east coast, and the Gulf Coast offset some underpredictions, resulting in smaller mean biases but a similar RMSE for the model compared to METAR as opposed to that compared to CASTNET. The difference between observed *T*2 from the two datasets is larger in cooler months than warmer months. The largest underpredictions occur in the spring (March, April, May – MAM) season. In general, GFSv15 underpredicts *T*2 for both CASTNET and METAR, consistent with cold biases found in other studies using GFSv15 (e.g., Yang, 2019). Such underpredictions will affect chemical forecasts, especially the forecast of O_3_. Consistent with the overall underpredictions of *T*2, GFSv15 overpredicts RH2 in general. The largest overprediction is found in spring (MBs of 3.4% and 2.7% with CASTNET and METAR, respectively), corresponding to the largest underprediction of *T*2 in spring (MBs of −0.5 and −0.4 °C with CASTNET and METAR, respectively). GFSv15 shows moderately good performance when predicting wind. The annual MB and NMB of WS10 compared to METAR are 0.4 m s^−1^ and 10.7%, respectively. A larger overprediction of WS10 is found with CASTNET than with other datasets (Zhang et al., 2016). GFSv15–CMAQv5.0.2 also gives higher overpredictions for CASTNET compared to METAR. The largest biases in wind speed are found in summer. GFSv15–CMAQv5.0.2 gives the largest cold biases and wet biases in spring, indicating the necessity of improving model performance in such seasons in future GFS–FV3 development.

By adopting the threshold of ≥ 0.1 mm h^−1^, performance compared to the CASTNET and METAR shows similar results: a large underprediction in hourly precipitation. Predicted monthly accumulated precipitation shows consistency in spatial distribution with observations from CCPA and GPCP (Fig. S3 in the Supplement). The high precipitation in the southeast is captured well in spring, while the high precipitation in the Midwest and south is captured well in other seasons. It indicates that GFSv15–CMAQv5.0.2 has good performance in capturing the spatial distributions of accumulated precipitation but has poor performance in predicting hourly precipitation. The precipitation from the original FV3 outputs is recorded as 6 h accumulated precipitation. Artificial errors were introduced to the forecast by an issue in precipitation preprocessing during the early stage of the development of the GFSv15–CMAQv5.0.2 system. The precipitation at the first hour of the 6 h cycle would be dropped occasionally. We corrected this issue and the hourly precipitation still shows a large underprediction compared to surface monitoring networks (Fig. S4 in the Supplement). It indicates the difficulty for the forecast system in capturing the temporal precipitation, especially during summer. During the summer season, the discrepancy in capturing the short-term heavy rainfall worsens the model performance in predicting hourly precipitation. Besides, we use the threshold of 0.1 mm h^−1^ to filter the valid records. If the model predicts precipitation that did not occur, the record will be excluded from the statistics calculation. However, all the predicted precipitation is counted in the spatial evaluation against the ensemble datasets of GPCP and CCPA. Therefore, the spatial performance of monthly accumulated precipitation shows better agreement than its of hourly statistics.

**Figure 1.**
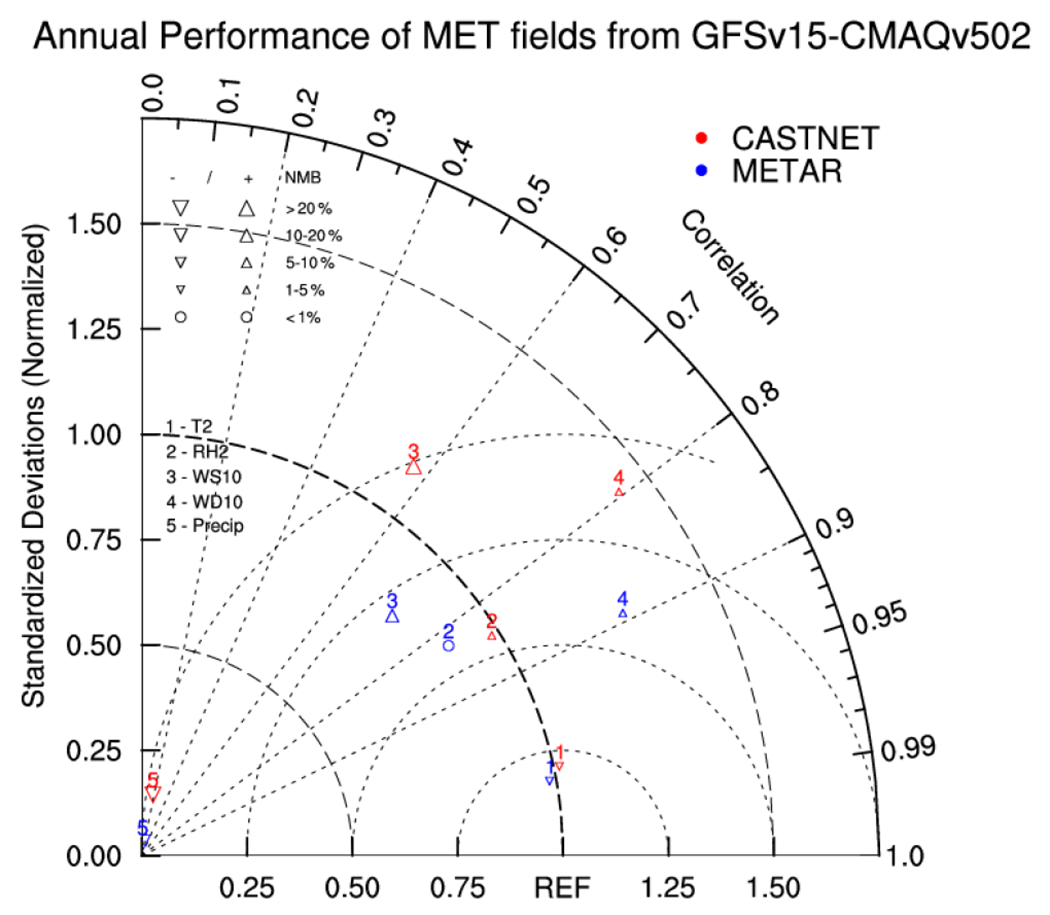
Taylor diagram (Taylor, 2001) with normalized standardized deviations (NSDs), Corr, and NMB for meteorological variables (*T*2, RH2, WS10, WD10, and Precip) compared to the CASTNET and METAR datasets. The REF marker at the *x* axis represents the desired performance. The closer each variable is to the REF marker, the better a performance the forecast system has for that variable.

An overall comparison of performance with the CASTNET and METAR datasets is performed using a Taylor diagram (Fig. 1). The NSDs, Corrs, and NMBs are considered. The NSDs are ratios of the variance of predicted values to the variance of observed values, following the equations by Wang et al. (2015). The NSDs represent the amplitude of variability. With the NSDs closer to 1, the predicted values have closer variance than the observed values. Consistent with other analysis in this section, larger biases and lower correlation in model wind speed and wind direction are found for CASTNET compared to METAR. The amplitude of variability of WS10 compared to CASTNET is overpredicted (with the NSD larger than 1), while it is underpredicted compared to METAR. Because of the postprocessing smearing of hourly precipitation, the variance of predicted precipitation is smaller than the observed one, leading to very small NSDs for precipitation. The location of the *T*2 and RH2 points near the REF marker in the Taylor diagram indicates that the GFSv15–CMAQv5.0.2 captures the magnitude and variability of these variables well.

### 3.2 Overall performance of chemical forecast over the CONUS

The performance of chemical forecasts (i.e., O_3_ and PM_2.5_) is evaluated on monthly, seasonal, and annual timescales for the studied period of 2019. The performance of the MDA8 O_3_ and the 24 h average PM_2.5_ (24 h average PM_2.5_) is regarded as the primary objective. Categorical performance evaluations for MDA8 O_3_ and 24 h average PM_2.5_ are also conducted. Table 3 shows the discrete statistics of predicted MDA8 O_3_ and 24 h average PM_2.5_ compared to AIRNow.

**Figure 2.**
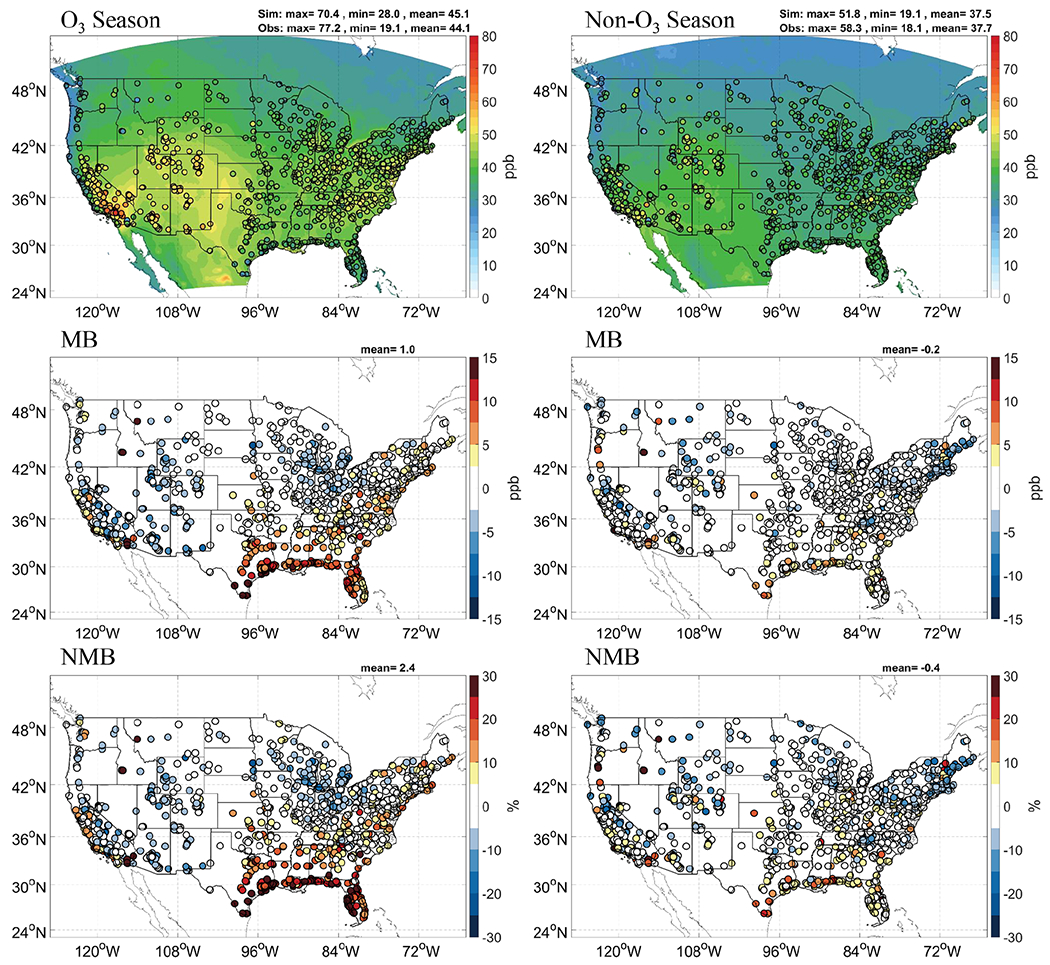
Spatial distribution of forecasted MDA8, MB, and NMB during the O_3_ and non-O_3_ seasons. Observation from AIRNow is shown as filled circles in the overlay plots of concentrations.

The GFSv15–CMAQv5.0.2 has good performance for MDA8 O_3_ on a seasonal and annual basis with MBs ≤ ±1.0 ppb, NMB ≤ 2.5%, and NME ≤ 20%. The monthly NMBs/NMEs are within ±15%/25%, respectively. Slight overpredictions and underpredictions are found in both seasons with MB of 1.0 and −0.2 ppb. The largest underprediction is found in spring months, especially in March. The underprediction of MDA8 O_3_ in spring months is consistent with the largest underprediction of *T*2 in spring. It indicates biases in predicted *T*2 could be one of the reasons for the corresponding biases in O_3_ prediction. Predicted MDA8 O_3_ is lower than observed values in major parts of the Midwest and western regions during the O_3_ season (Fig. 2), which is consistent with an underprediction of *T*2 in summer. But GFSv15–CMAQv5.0.2 gives very high O_3_ in the southeastern US, especially in areas near the Gulf Coast. Such overpredictions compensate for moderate underpredictions in the Midwest and west, causing an overall overprediction in the overall CONUS. In the non-O_3_ season, GFSv15–CMAQv5.0.2 can forecast the spatial variations of MDA8 O_3_ well with overall underpredictions in the northeast.

**Figure 3.**
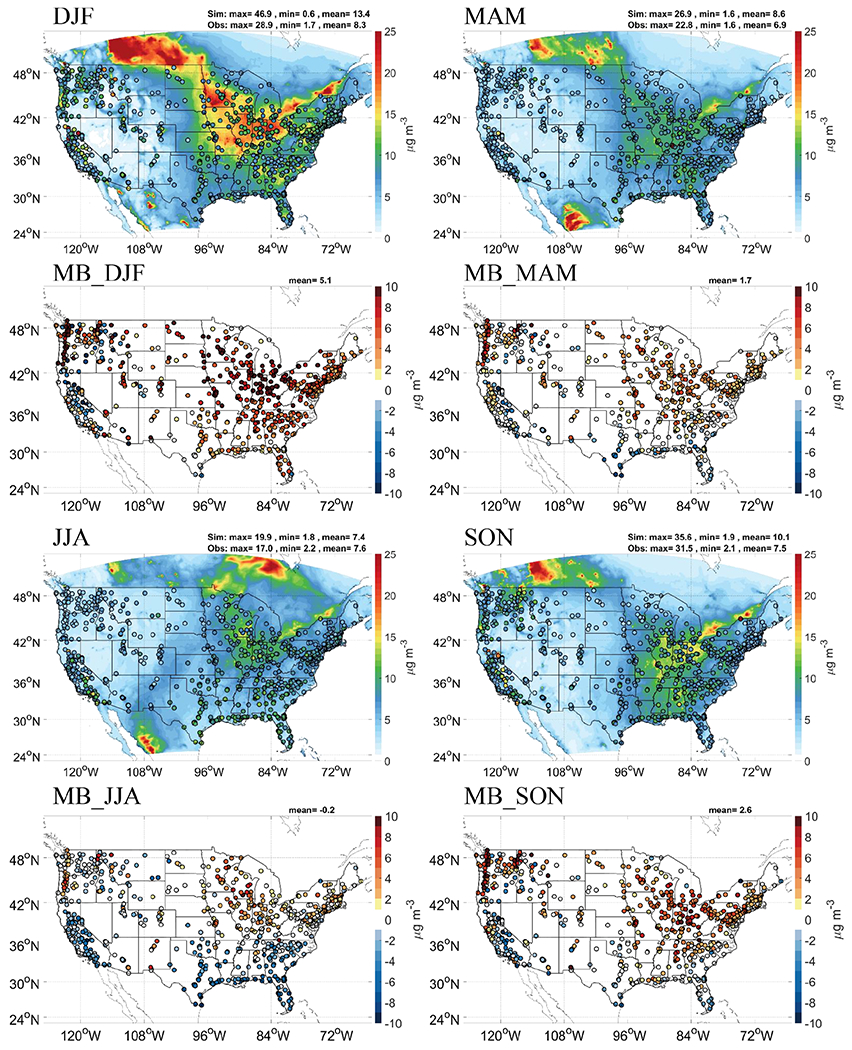
Forecasted seasonal daily PM_2.5_ by GFSv15–CMAQv5.0.2 overlaid observations from AIRNow and MB compared to observations from AIRNow.

Unlike the good performance for O_3_, GFSv15–CMAQv5.0.2 gives significant overpredictions for 24 h average PM_2.5_ with annual MB, NMB, and NME of 2.2 μg m^−3^, 29.0%, and 65.3%, respectively (Table 3). The MBs and NMBs range from −0.2 to 5.0 μg m^−3^ and −2.6% to 59.7% across the four seasons. With the exception of California and the southeast, predicted 24 h average PM_2.5_ shows overprediction during most of the year in spring, autumn, and winter (Fig. 3). Moderate underpredictions of PM_2.5_ are found in California during spring, autumn, and summer and are found in the southeast during summer. Using the historical emission inventories from NEI 2005 and NEI 2014 instead of the latest version of NEI 2017 is one of the reasons for the overpredictions of PM_2.5_ concentrations in 2019. The significant overprediction mainly occurs in the northern regions during cooler months, indicating it is underlaid by systematic biases. The annual emission of primary PM_2.5_ and coarse-mode PM (PMC) are shown in Fig. S5 in the Supplement. As an important surrogate for the fugitive dust, the spatial distribution of large PMC emission is associated with the regions which have the significant overprediction in cooler months. In reality, the meteorological conditions could greatly impact the amount and characteristics of anthropogenic fugitive dust. For example, the snow cover and the soil moisture are important factors in calculating the dust emissions in SMOKE. However, the anthropogenic fugitive dust implemented in this GFSv15–CMAQv5.0.2 system was not adjusted by the precipitation and snow cover. It will lead to a significant overestimation in the anthropogenic dust emission. The impact of the meteorological factor on anthropogenic fugitive dust emission and the PM_2.5_ prediction will be further discussed in Sect. 4.

**Figure 4.**
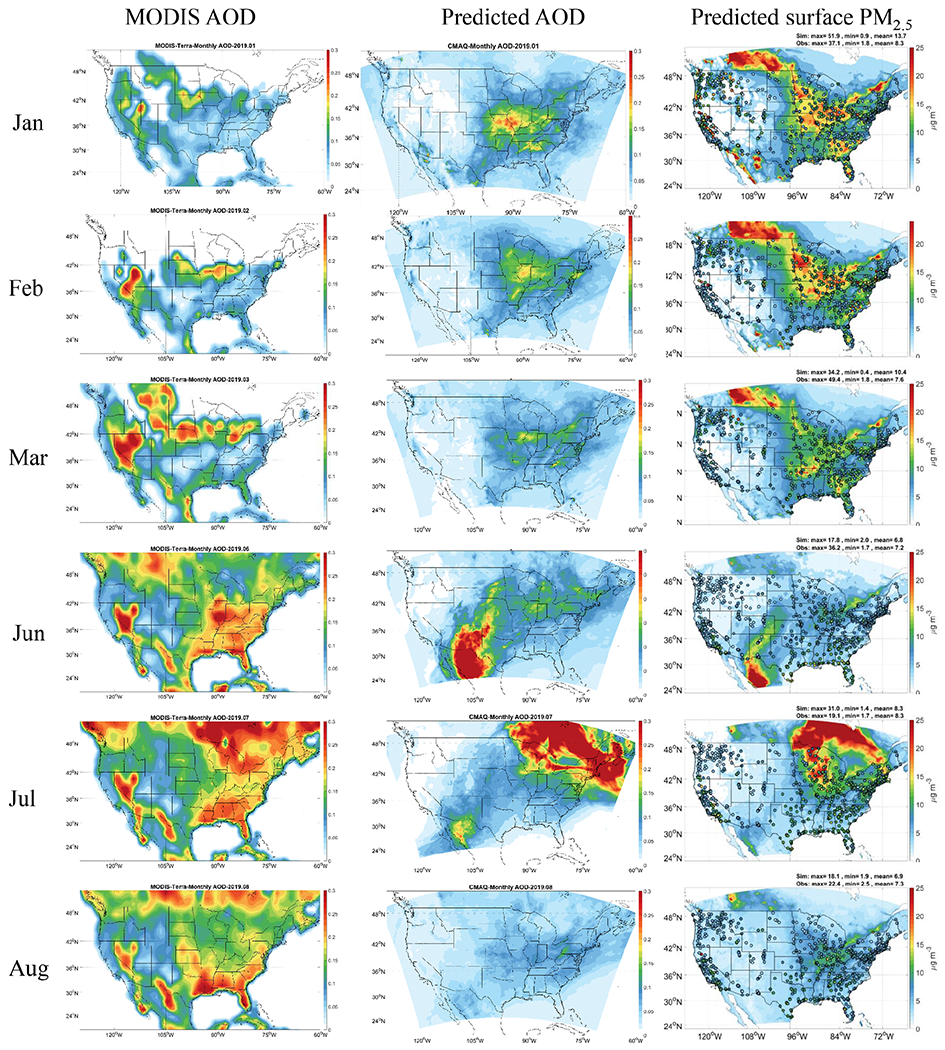
Monthly AOD from MODIS (left), predicted AOD from GFSv15–CMAQv5.0.2 (middle), and predicted surface 24 h average PM_2.5_ (right).

Murphy et al. (2017) found that secondary organic aerosols (SOAs) generated from anthropogenic combustion emissions were important missing PM sources in California prior to CMAQv5.2. The largest underpredictions of PM_2.5_ occur in the southeast in summer. Biogenic volatile organic compounds (BVOCs) and biogenic SOA (BSOA) are most active in the southeast region in summer. Many missing sources and mechanisms for SOA formation from BVOCs have been identified in recent years (Pye et al., 2013, 2015, 2017; Xu et al., 2018) and have resulted in significant improvements in predicting SOA in the southeast using CMAQv5.1 through v5.3. Anthropogenic emissions and aerosol inorganic compounds were found to have impacts on BSOA (Carlton et al., 2018; Pye et al., 2018, 2019). Such interactions and mechanisms are not represented sufficiently in CMAQv5.0.2, further enhancing the biases in predicted PM_2.5_ in the southeast. The evaluation of predicted AOD compared to observations from MODIS is shown in Fig. 4. High predicted AOD in the Midwest during cooler months shows consistency with MODIS and corresponds to high surface PM_2.5_ predictions. High predicted AOD is missing in California, corresponding to the underprediction of surface PM_2.5_ in California. In summer months, AOD is greatly underpredicted in California and the southeast, which may be caused by the previously mentioned missing sources of SOA.

### 3.3 Categorical evaluation

**Figure 5.**
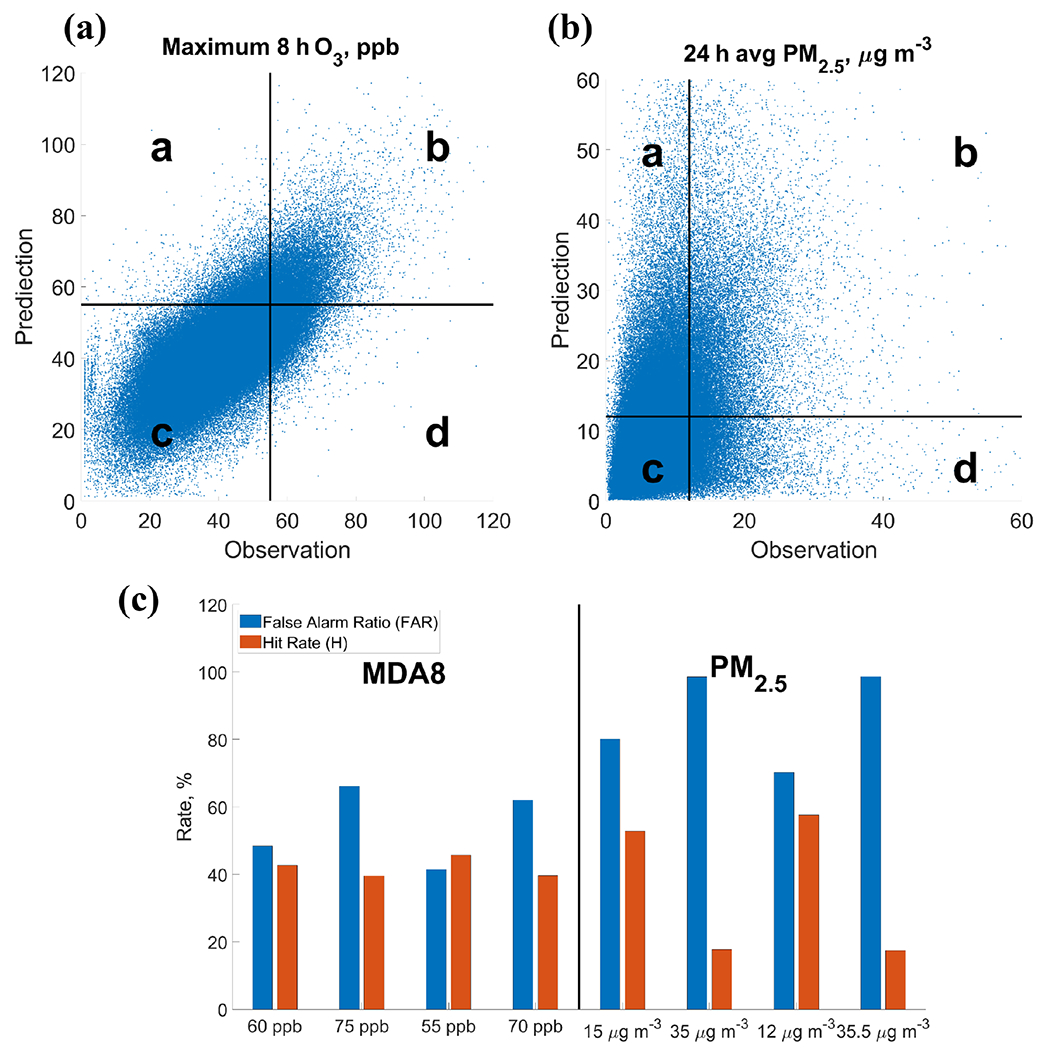
Categorical evaluation of MDA8 and 24 h average PM_2.5_: (**a**) scatterplot of predicted and observed MDA8; the scatters are divided into four areas using the threshold of 55 ppb for both observation and prediction. (**b**) Scatterplot of predicted and observed 24 h average PM_2.5_; the scatters are divided into four areas using the threshold of 12 μg m^−3^ for both observation and prediction. (**c**) False alarm ratio (FAR) and hit rate (*H*) in four categories for forecasts of MDA8 and 24 h average PM_2.5_.

A categorical evaluation is conducted to quantify the accuracy of the GFSv15–CMAQv5.0.2 system in predicting events in which the air pollutants exceed moderate or unhealthy categories for the US AQI (http://www.airnow.gov, last access: 10 August 2020). The scatterplots for predicted and observed MDA8 O_3_ and 24 h average PM_2.5_ are shown in Fig. 5a and b, respectively. Numbers of the scatters in the four areas (a) to (d) are indicated in the Eqs. (1) and (2) in Sect. 2.2. The higher the FAR is, the more GFSv15–CMAQv5.0.2 overpredicts the AQI leading to false air quality warnings. The higher the *H* is, the more successfully the exceedances are captured by the GFSv15–CMAQv5.0.2 system. In this study, the thresholds for the two categories of “Moderate” and “Unhealthy for sensitive groups” are considered. Since 2018, they are defined as 55 and 70 ppb for MDA8 O_3_ and 12 and 35.5 μg m^−3^ for 24 h average PM_2.5_. For comparison with previous studies, the historical thresholds are also included in the evaluation: 60 and 75 ppb for MDA8 O_3_ and 15 and 35 μg m^−3^ for 24 h average PM_2.5_. The metrics in four categories, corresponding to four thresholds, are shown in Fig. 5c. Categorical performance under stricter AQI standards is better than under historical standards. For example, the FAR decreases from 48.4% to 41.4%, and the *H* increases from 42.7% to 45.8% with the Moderate threshold change from 60 to 55 ppb. This could be due to the better performance of the forecast system for values closer to the annual average level (~ 40 ppb). The scatters are more discrete for extreme values. When the thresholds of MDA8 O_3_ are closer to the average level, the categorical performance increases. A similar improvement in the FAR and *H* for predicting categorical 24 h average PM_2.5_ can be found when the threshold changes from 15 to 12 μg m^−3^: the FAR decreases from 80.1% to 70.3%, and the *H* increases from 52.8% to 57.6%. However, the FAR is high (over 90%) and the *H* is much lower under the threshold of 35.5 μg m^−3^. It is because most of the false alarms occur when observed 24 h average PM_2.5_ is lower than 20 μg m^−3^ and the predicted values are higher than 20 μg m^−3^. It shows the poorer performance in correctly capturing the category of Unhealthy for sensitive groups due to the significant overprediction of PM_2.5_ in cooler months.

Major RT-AQF systems over the world were comprehensively reviewed in Zhang et al. (2012a, b). Here we include a comparison with more recent air quality forecasting studies. Table S3 summarizes air quality forecasting skills reported in the literature from assessments of other air quality forecasting studies from Canada (Moran et al., 2018; Russell et al., 2019), Europe (Struzewska et al., 2016; D’Allura et al., 2018; Podrascanin, 2019; Spiridonov et al., 2019; Stortini et al., 2020), East Asia (Lyu et al., 2017; Zhou et al., 2017; Peng et al., 2018; Ha et al., 2020), and CONUS (Kang et al., 2010; Zhang et al., 2016; Lee et al., 2017), along with that from this work. For those studies with data assimilation in air quality forecasting, the performance from the raw results without data assimilation is presented. The performance in predicting O_3_ and PM vary greatly between model systems. The discrete and categorical performance in O_3_ prediction is not significantly better than that in PM prediction. O_3_ tends to be slightly overpredicted on an annual basis or for the warmer months. The annual NMB and Corr for O_3_ over the North America are 1.4% and 0.76 for 2010 in Moran et al. (2018), while they are 1.0% and 0.73 in this study. However, the performance in PM_2.5_ prediction varies greatly from our study. The PM_2.5_ for warmer months was moderately overpredicted in Russel et al. (2019), with the MBs ranging from 3.2 to 5.5 μg m^−3^. The categorical performance of GFSv15–CMAQv5.0.2 in predicting MDA8 O_3_ is similar to that of the previous NAQFC (Kang et al., 2010), in which the FAR and *H* are ~ 68% and ~ 31% for the Unhealthy for sensitive groups category, and the *H* is ~ 47% for the Moderate category. The *H* for PM_2.5_ also decreased greatly from ~ 46% for the Moderate category to ~ 21% for the Unhealthy for sensitive groups category, and the FAR was over 90% for the Unhealthy for sensitive groups category in Kang et al. (2010). The overpredicted PM_2.5_ was also found when using the historical 2005 NEI in a forecast for January 2015 (Lee et al., 2017). The performance was improved by updates of 2011 NEI and real-time dust and wild fire emissions. It indicates the need to improve our emission inventory. As for the categorical performance in regions other than CONUS, the air quality standards vary (Oliveri Conti et al., 2017). For example, the National Ambient Air Quality Standards (NAAQSs), the Ambient Air Quality and Cleaner Air for Europe (CAFE) Directive (2008/50/EC), and the national ambient air quality standard (GB 3095-2012) are set up by the US, Europe, and China, respectively. Metrics also vary between studies. The primary forecasting products are O_3_ and PM_10_ from some forecasting systems instead of O_3_ and PM_2.5_ in this study. The threshold for a categorical evaluation of O_3_ used in D’Allura et al. (2018) was 83.0 μg m^−3^. The applied metrics of the false alarm ratio and probability of detection (POD) were defined the same as the FAR and *H* used in our study. The FAR and POD were 36.14% and 71.16%, respectively. The categorical evaluation of PM_2.5_ in Ha et al. (2020) was applied for four categories: (1) 0–15 μg m^−3^, (2) 16–50 μg m^−3^, (3) 51–100 μg m^−3^, and (4) > 100 μg m^−3^. The overall FAR and detection rate for the four categories are 59.0% and 36.1%, respectively. Although the metrics of the FAR and detection rate were defined for the four categories, rather than for every single category as in this study, the categorical performance is comparable with our results. In general, the discrete and categorical performance of O_3_ forecast in this study is comparable to that of the air quality forecasting systems in many regions of the world. However, the PM forecasts vary greatly between studies. While our GFSv15–CMAQv5.0.2 system shows consistent performance with the systems covering CONUS, the high FAR and low *H* for the Unhealthy for sensitive groups category with higher thresholds indicate that the categorical performance could be further improved by addressing the significant overprediction during cooler months in this study.

### 3.4 Region-specific evaluation

As discussed in Sect. 3.2, biases in predicted O_3_ and PM_2.5_ vary from region to region. To further analyze the region-specific performance of the GFSv15–CMAQv5.0.2 system, an evaluation for 10 regions within CONUS is conducted. By identifying the detailed characteristics of region-specific biases and indicating the underlying causes for such biases, this section aims to help the NAQFC to improve its forecast ability for specific regions.

**Figure 6.**
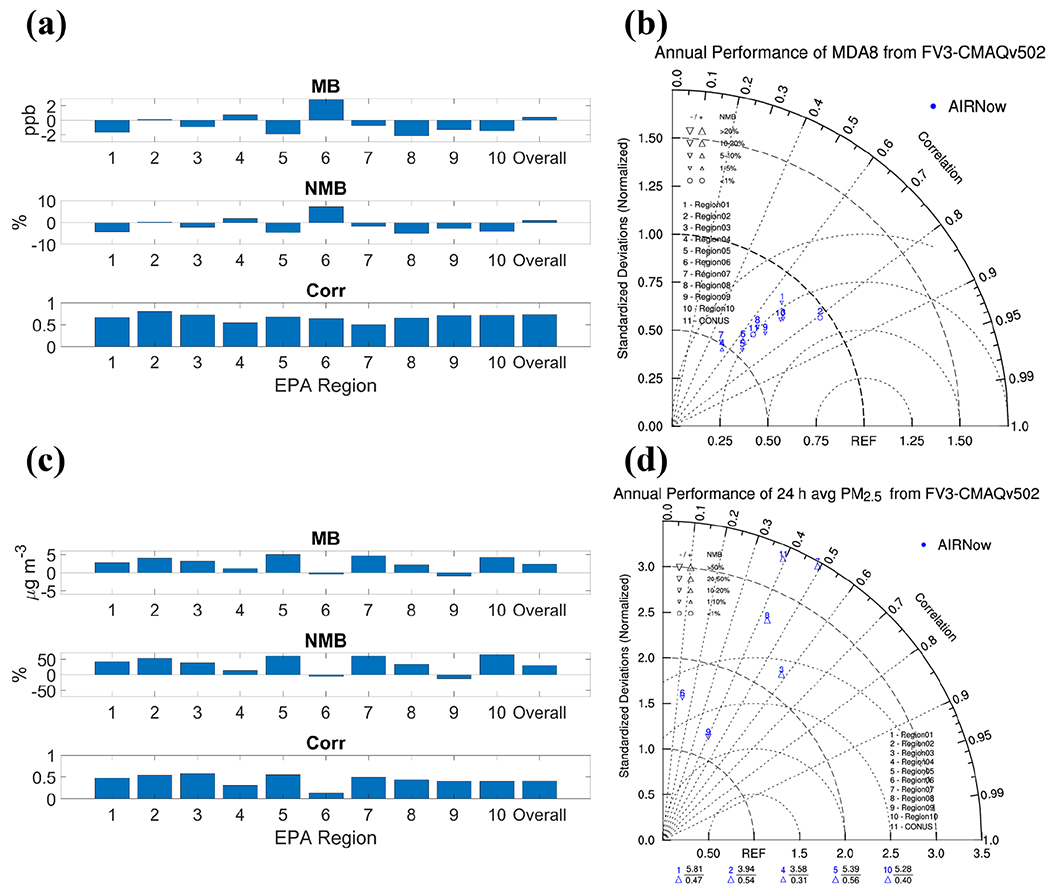
Annual performance of MDA8 in 10 CONUS regions (**a**); Taylor diagram for annual performance of MDA8 (**b**); annual performance of 24 h average PM_2.5_ in 10 CONUS regions (**c**); Taylor diagram for annual performance of 24 h average PM_2.5_. Outliers represent regions with NSDs > 3.5 (**d**).

Figure 6 shows the annual model performance for MDA8 O_3_ and 24 h average PM_2.5_ in the 10 CONUS regions. In Sect. 3.2, a slight underprediction of MDA8 O_3_ on an annual basis was found over the CONUS. MDA8 O_3_ is underpredicted in most of the regions except regions 2, 4, and 6 (Fig. 6a). The overpredictions in regions 4 and 6 are mostly from the large biases near the coast area during the O_3_ season. Correlations between predictions and observations in most of the regions are higher than 0.6, except for 0.55 in region 4 and 0.50 in region 7. Poor performance in regions 4 and 7 is illustrated by the Taylor diagram (Fig. 6b). Small Corr and NSD result in the markers of regions 4 and 7 lying farthest from the reference point. The amplitude of variability of the predicted MDA8 O_3_ is smaller than the observed values in all the regions, especially in regions 4 and 7. The performance in region 2 is the best, with the smallest MB or NMB, the highest Corr, and similar variability in predictions and observations. The time series of the MDA8 O_3_ for the 10 regions during 2019 is shown in Fig. S6 in the Supplement. Regions 1, 2, 4, and 6 show different results for the O_3_ season and the non-O_3_ season: GFSv15–CMAQv5.0.2 tends to overpredict MDA8 O_3_ during the O_3_ season and underpredicts it during the non-O_3_ season. The underprediction during spring months, which is indicated in Sect. 3.2, can be also found in most of the regions with obvious gaps between observed and predicted curves in March and April. The lowest O_3_ predictions occur at 05:00 local standard time (LST) in most of the regions (Fig. S7 in the Supplement). For regions 4 and 6, significant overprediction occurs not only during the O_3_ season for MDA8 O_3_ (which mainly occurs during the daytime) but also during the nighttime. During the non-O_3_ season, the biases in predicting MDA8 O_3_ for regions 4 and 6 are small and consistent with good daytime predictions. However, O_3_ is still overpredicted during the nighttime in these regions, associated with the collapse of the boundary layer and difficulty in simulating its time and magnitude (Hu et al., 2013; Cuchiara et al., 2014; Pleim et al., 2016).

Consistent with the analysis in Sect. 3.2, PM_2.5_ is significantly overpredicted in most of the regions except in regions 4, 6, and 9 (Fig. 6c). The underprediction during warmer months, likely due to missing sources and mechanisms for BSOA, compensates for the annual biases in regions 4 and 6, leading to smaller MBs or NMBs but low correlations in these regions. The variability in predictions is much larger than in observations, with the NSDs > 1 for all regions (Fig. 6d). The forecast system has the best performance in region 9 with an NSD of 1.2, an NMB of −12.0%, and a Corr of 0.40. Figure S8 in the Supplement shows the time series of 24 h average PM_2.5_ in the 10 CONUS regions. The gaps between observed and predicted curves are large in cooler months, but the GFSv15–CMAQv5.0.2 system has relatively good performance in warmer months for most of the regions. Less overprediction is found in regions 6 and 9 during cooler months, and those regions generally show the best performance (see Taylor diagram). The different biases across the regions further indicate that multiple factors likely contribute to them.

## 4 Discussion

### 4.1 Meteorology–chemistry relationships

We further quantify the meteorology–chemistry relationships by conducting the region-specific evaluation of the meteorological variables. The regional performance for the major variables is shown in Fig. S9 in the Supplement. The regional biases in *T*2 predictions show high correlation with the regional biases in MDA8 O_3_. It indicates that the cold biases in the Midwest (including region 5) and the warm biases near the Gulf coast (including regions of 4 and 6) are important factors for the O_3_ underprediction and overprediction in those regions, respectively. The O_3_–temperature relationship was found (Sillman and Samson, 1995; Sillman, 1999). O_3_ is expected to increase with increasing temperature within a specific range of temperature (Bloomer et al., 2009; Shen et al., 2016). The surface MDA8 O_3_–temperature relationship was found at approximately 3–6 ppb K^–1^ in the eastern US (Rasmussen et al., 2012). According to such relationships, the biases in *T*2 predictions could explain a large portion of the O_3_ biases. Heavy convective precipitation and tropical cyclones have a large impact in the southeastern US, which covers mainly regions 4 and 6. Therefore, the performance in precipitation predictions is lower in those two regions compared to other regions as we discussed regarding the model performance in capturing short-term heavy rains during summer seasons in Sect. 3.1. Meanwhile, the performance in wind predictions in regions 4 and 6 is relatively poor. Such performance in the meteorological predictions is consistent with the mixed performance in PM_2.5_ prediction in regions 4 and 6. The low temporal agreement shown as correlations of predicted PM_2.5_ in those two regions can be attributed to the discrepancy in meteorological inputs, mainly in precipitation and wind.

### 4.2 Major biases in O_3_ predictions

Prediction and simulation of O_3_ in coastal or marine areas are impacted by halogens chemistry and emissions (Adams and Cox, 2002; Sarwar et al., 2012; Liu et al., 2018), including bromine and iodine chemistry (Foster et al., 2001; Sarwar et al., 2015; Yang et al., 2020) and oceanic halogen emissions (Watanabe, 2005; Tegtmeier et al., 2015; He et al., 2016). CMAQv5.0.2 only has simple chlorine chemistry for CB05 mechanisms, and the reduction of O_3_ by reaction with bromine and iodine is not included in CMAQv5.0.2. Iodide-mediated O_3_ deposition over seawater and detailed marine halogen chemistry has been found to reduce O_3_ by 1–4 ppb near the coast (Gantt et al., 2017), suggesting that the missing halogen chemistry and O_3_ deposition processes contribute to overpredicted O_3_ in coastal and marine areas seen here. Coastal and marine areas are also impacted by air–sea interaction processes, which are simply represented in the current meteorological models without coupling oceanic models (He et al., 2018; Y. Zhang et al., 2019a, b). For example, coastal O_3_ mixing ratios are impacted by predicted sea surface temperatures and land–sea breezes through their influence on chemical reaction conditions and diffusion processes. As discussed in Sects. 3.1 and 4.1, the GFSv15–CMAQv5.0.2 system has poorer performance in predicting the meteorological variables in regions of 4 and 6, which could contribute to biases in O_3_ predictions directly or indicate missing land–sea breezes and thus missing transport effects in the GFSv15–CMAQv5.0.2 air quality forecasting system.

In addition to the impact of meteorological biases and missing halogen chemistry on the O_3_ overprediction near the Gulf coast, the overestimated volatile organic compound (VOC) emission could enhance the O_3_ biases. The anthropogenic VOC emissions continuously decrease from historical NEIs to the 2016 NEI (http://views.cira.colostate.edu/wiki/wiki/10202/inventory-collaborative-2016v1-emissions-modeling-90platform, last access: 10 October 2020). We compare the VOC emissions between the 2016 NEI and the emissions used in this study. The difference in the elevated source of pt_oilgas is shown in Fig. S10 in the Supplement. The Gulf coast is impacted by the oil and gas sector due to the oil and gas fields and the exploration activity near it. By comparing the newer NEI to the current NEI we used in the system, we found that the overestimation of the VOCs could be one aspect of the O_3_ overprediction near the Gulf Coast because we only project the SO_2_ and NO*_x_* from the 2005 NEI to 2019 but we do not project the VOCs for the elevated sources. The monthly VOC emissions from the pt_oilgas sector for July in regions 4 and 6 are 2876.0 tmonth^−1^, while they are 2497.0 tmonth^−1^ in the 2016 NEI. The reduction is mainly located along the coastline, where the significant overprediction takes place. It indicates the complicated effect of meteorological biases, missing gas-phase chemistry, and the overestimation of emissions on the O_3_ prediction in these regions.

**Figure 7.**
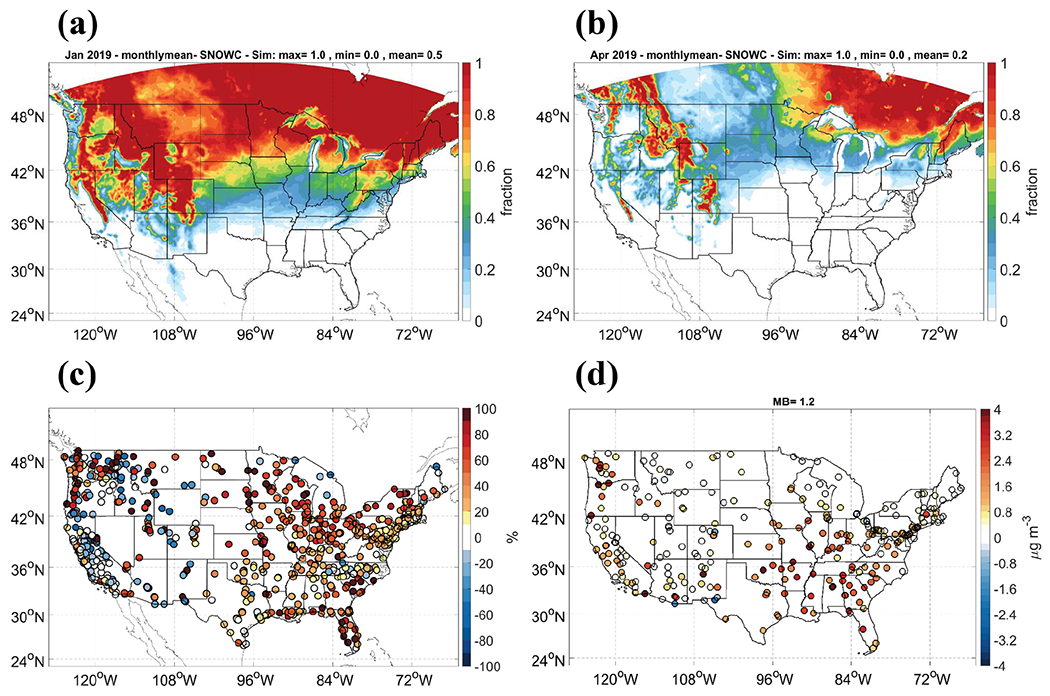
The predicted average snow cover for (**a**) January and (**b**) April. (**c**) The difference in NMBs of PM_2.5_ by adjusting PM emission for January. Positive values stand for improvement in biases with NMBs closer to 0. (**d**) MBs in PM_2.5_ soil composition with adjustment of PM emission for January.

The O_3_ concentration is underpredicted for the northeast, mid-Atlantic, Midwest, mountainous states, and northwest (mainly corresponding to the regions 1, 3, 5, 8, and 9) during the non-O_3_ season. A large difference in dry-deposition algorithms between CMAQv5.0.2 and other common parameterizations was reported (Park et al., 2014; Wu et al., 2018). A large discrepancy between modeled dry-deposition velocity of O_3_ by CMAQv5.0.2 and the observation during winter was shown and attributed to the deposition to snow surface. An improvement was indicated in revising the treatment of deposition to snow, vegetation, and bare ground in CMAQv5.0.2. Lower deposition to snow was found to improve the consistency between the O_3_ deposition modeled by CMAQv5.0.2 and the observations. Therefore, the dry-deposition module in v5.0.2 needs to be updated and improved for more accurate representation of low-moderate O_3_ mixing ratios (Appel et al., 2021). For the cases in this study, the predicted snow cover for the months of January and April in winter and spring are shown in Fig. 7a and b. The underpredicted O_3_ during the non-O_3_ season may be caused by the overestimated O_3_ deposition to snow in the northern regions, corresponding to the previous regions 1, 3, 5, 8, and 9. The mixed effects of the temperature–O_3_ relationship discussed above and the large deposition to snow contribute to the moderate O_3_ underpredictions.

### 4.3 Major biases in PM_2.5_ predictions

**Figure 8.**
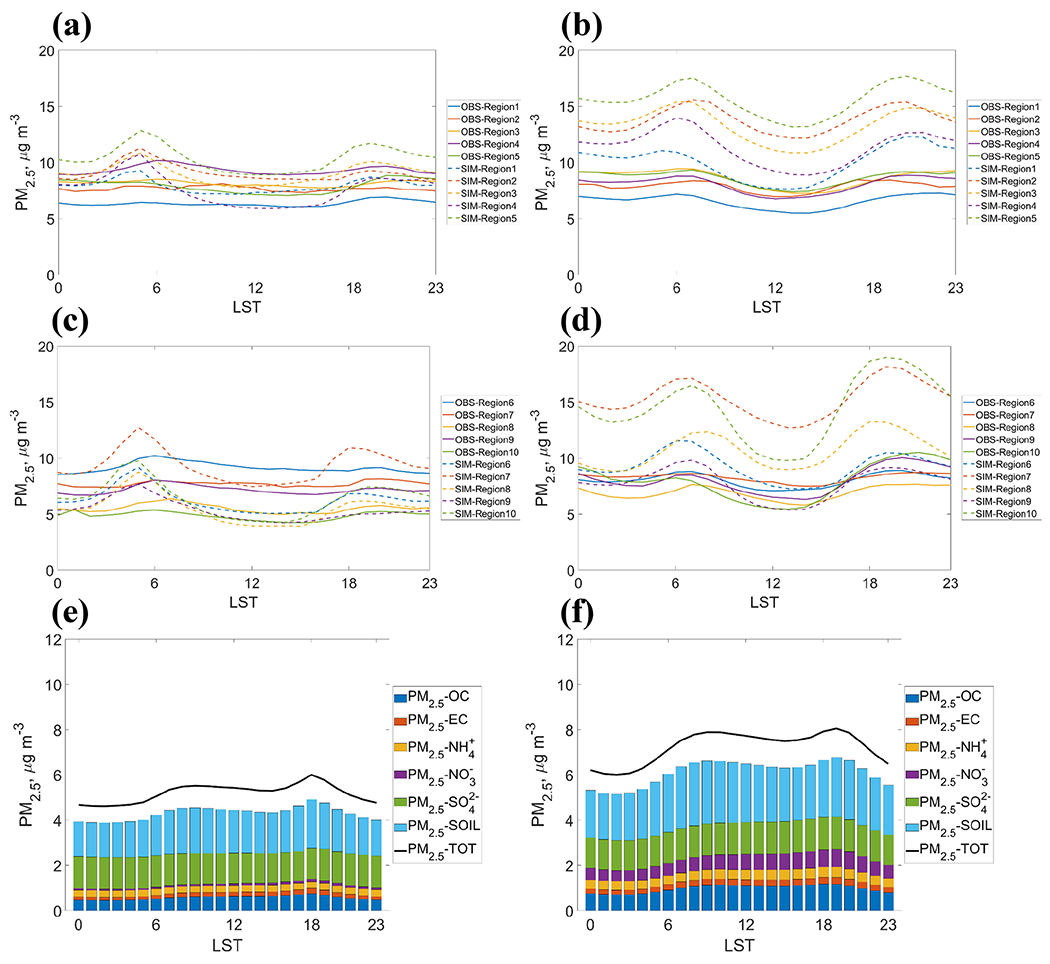
Diurnal PM_2.5_ in (**a**) the O_3_ season for regions 1 to 5; (**b**) the non-O_3_ season for regions 1 to 5; (**c**) the O_3_ season for regions 6 to 10; (**d**) the non-O_3_ season for regions 6 to 10. Solid curves are observed values and dashed curves are predicted values. Average of predicted PM_2.5_ and components of PM_2.5_ within CONUS in (**e**) the O_3_ season and (**f**) the non-O_3_ season.

Major biases in PM_2.5_ prediction are distinguished for warmer and cooler months in Sect. 3. To further analyze the underlying causes for varied patterns and performance on a season- and region-specific basis, diurnal evaluations for PM_2.5_ and chemical components of PM_2.5_ during the O_3_ season and the non-O_3_ season are shown in Fig. 8. GFSv15–CMAQv5.0.2 has a large seasonal variation in diurnal PM_2.5_, inconsistent with the observation. While PM_2.5_ is underpredicted during daytime in regions 4, 6, 8, and 9 during the O_3_ season, PM_2.5_ is always overpredicted across the day during the non-O_3_ season except for region 9. Increased organic carbon (OC), particulate nitrates, soil and unspecified coarse-mode components contribute to most of the increase in predicted total PM_2.5_. The general cold biases over CONUS, especially in region 5, could make the GFSv15–CMAQv5.0.2 system predict higher nitrate particulates, leading to a larger increase in PM_2.5_ from the O_3_ season to the non-O_3_ season. Emissions vary from month to month in the year (Fig. S11a in the Supplement). There are larger emissions for NH_3_, NO*_x_*, VOC, primary coarse PM, and primary PM_2.5_ in the O_3_ season compared to the non-O_3_ season. Primary organic carbon (POC) emissions are higher in the O_3_ season. Changes in emissions are not fully consistent with the changes in PM_2.5_ components, indicating that other biases or uncertainty could also contribute to the significant overprediction during the non-O_3_ season. For example, the implementation of a bidirectional flux of NH_3_ and the boundary layer mixing processes under more stable conditions (during the non-O_3_ season) in the GFSv15–CMAQv5.0.2 system need to be further studied. Pleim et al., (2013, 2019) found that the NH_3_ fluxes and concentrations could be better simulated and the monthly variations in NH_3_ concentrations were larger compared to the raw model by implementing the bidirectional flux of NH_3_. The absolute biases for diurnal PM_2.5_ are generally larger during nighttime in most of the regions, except for region 9. This is consistent with the analysis by Appel et al. (2013), which suggested that the efforts of improving nighttime mixing in CMAQv5.0 are further needed, further indicating the need for improvements of CMAQ in predicting dispersion and mixing of air pollutants under stable boundary layer conditions. The forecast system gives the highest PM predictions at two peaks during the day: 06:00 and 19:00 in the O_3_ season and 07:00 and 20:00 in the non-O_3_ season at LST, respectively, corresponding to the shifting between day-light saving time and LST. The two diurnal peaks are caused by the diurnal pattern of emissions (Fig. S11b). PM is mostly emitted during the daytime from 06:00 to 18:00. With the development of the boundary layer during the daytime, surface PM_2.5_ concentrations will be reduced by the diffusion. During dawn and dusk, the boundary layer transits between stable and well-mixed conditions. The increased emission and secondary production of PM_2.5_ will be accumulated within the boundary layer, causing the high peaks during dawn and dusk.

**Figure 9.**
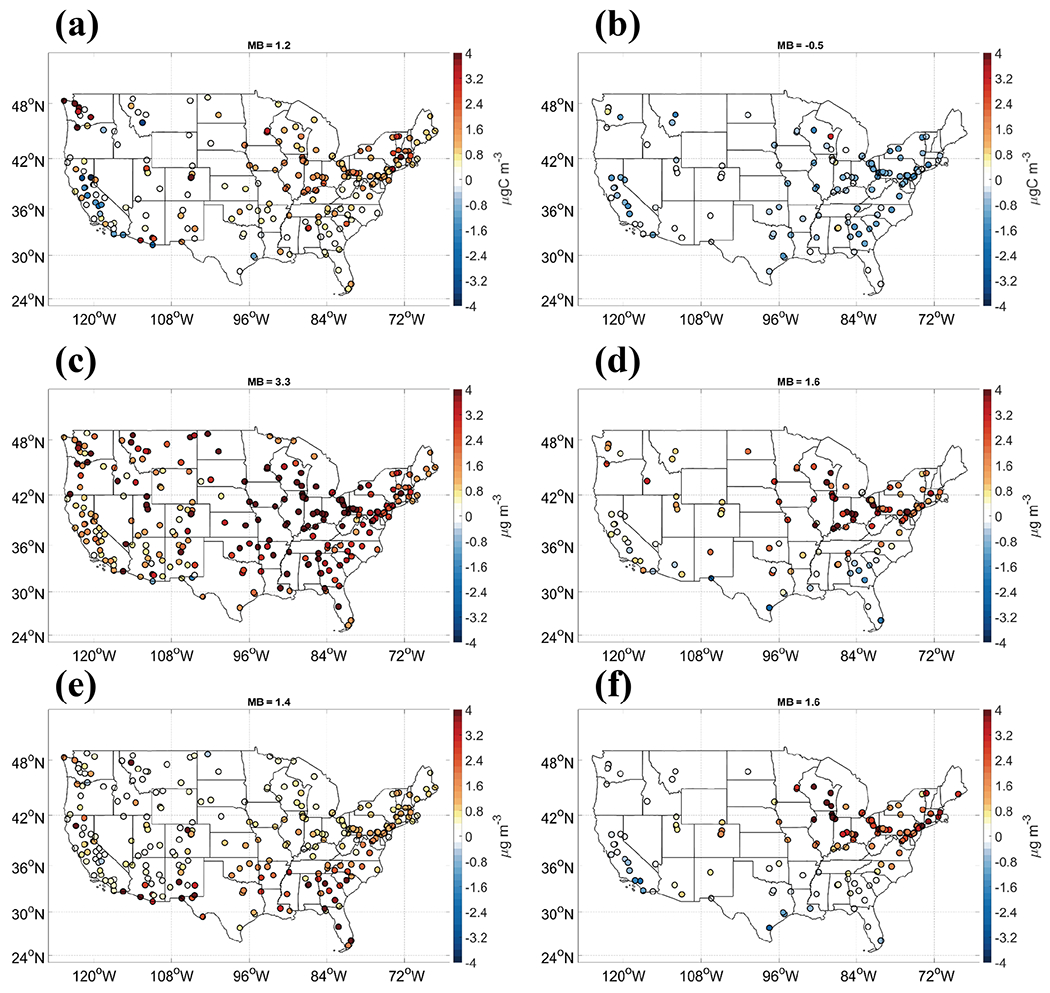
Mean biases in PM_2.5_ compositions: (**a**) OC for January, (**b**) OC for July, (**c**) SOIL for January, (**d**) SOIL for July, (**e**) sulfate for January, and (**f**) sulfate for July.

The variation in predicted PM_2.5_ composition between cooler and warmer months indicates that major seasonal biases are caused by multiple factors. We introduce the Air Quality System (AQS) dataset for the evaluation of daily PM_2.5_ composition to provide additional insight into the specific reasons. Figure 9 shows the biases of the key PM_2.5_ composition for the cooler month of January and warmer month of July. While the overall mean biases of PM_2.5_ composition, including elemental carbon (EC), ammonium (${\mathrm{NH}}_{4}^{+}$), and nitrate (${\mathrm{NO}}_{3}^{-}$), are within ±0.5 μg m^−3^ for all months of the year, the major biases in PM_2.5_ predictions are mostly contributed by OC, soil components (SOIL), and sulfate (${\mathrm{SO}}_{4}^{2-}$). The soil components are estimated using the Interagency Monitoring of Protected Visual Environments (IMPROVE) equation and specific constituents (Appel et al., 2013). During a cooler month, the significant overprediction in PM_2.5_ is mainly attributed to the overprediction in OC and SOIL. During warmer months, the overprediction of SOIL and sulfate compensates for the overall underprediction in OC in v5.0.2, leading to the moderate PM_2.5_ underprediction in the southeast but slight overprediction in the Midwest, mid-Atlantic, and the northeast. These high PM_2.5_ SOIL concentrations are consistent in spatial characteristics with large emissions of anthropogenic primary PM_2.5_ and primary coarse PM in the Midwest, northeast, and northwest. The underprediction in PM_2.5_ OC during summer compensates for the overestimation in dust during cooler months, resulting in the overall biases with an annual NMB of 30.0%.

The large emissions of anthropogenic primary coarse PM as well as the wind-blown dust are the major sources for predicted PM_2.5_ SOIL components. Appel et al. (2013) indicated CMAQ overpredicted soil components in the eastern United States partially due to the anthropogenic fugitive dust and wind-blown dust emissions. The overprediction in PM_2.5_ soil compositions by our forecast system could mainly be attributed to the overestimation of the anthropogenic fugitive dust emission because the meteorological conditions were not included in processing the anthropogenic fugitive dust sector. The dust-related components of aluminum, calcium, iron, titanium, silicon, and coarse-mode particles are overestimated in the regions with snow and precipitation, especially during winter, early spring, and late autumn with snow cover in the north, which contributes to the PM_2.5_ overprediction, with a more significant temporal–spatial pattern in the north US during cooler months.

An adjustment of precipitation and snow cover for fugitive dust was implemented in the operational NAQFC. The dust-related PM emissions will be cleaned up using a factor of 0.01 when the snow cover is higher than 25% or the hourly precipitation is higher than 0.1 mm h^−1^ before they are used as input for CMAQv5.0.2 forecast. We conduct a sensitivity simulation for January 2019 using the GFSv15–CMAQv5.0.2 system with the adjustment implemented in the operational NAQFC. Figure 7c shows that the PM_2.5_ overprediction in the northern regions 1, 2, 5, and 10 during January is greatly improved corresponding to the spatial–temporal characteristics of snow cover. The monthly MB and NMB for January improves from 5.5 μg m^−3^ and 66.9% to 2.1 μg m^−3^ and 24.0%, respectively. The improvement is mainly attributed to the decrease in overpredictions in PM_2.5_ soil components, with MBs decreased from 3.3 to 1.2 μg m^−3^ for January (Fig. 7d). The overprediction in the northeast and northwest during spring is expected to be improved by the suppression of the fugitive dust by the snow during early spring. This indicates the importance of including the meteorological forecast in processing the emission of anthropogenic fugitive dust. It should be calculated inline or be adjusted by the meteorological forecast.

In CMAQv5.0.2, the primary organic aerosol (POA) is processed as non-volatile. The emissions of semivolatile and intermediate-volatility organic compounds (S/IVOCs) and their contributions to the SOA are not accounted for in the aerosol module. In the recent versions of CMAQ, two approaches linked to POA sources have been implemented. One introduces semivolatile partitioning and gas-phase oxidation of POA emissions. The other one (called pcSOA) accounts for multiple missing sources of anthropogenic SOA formation, including potential missing oxidation pathways and emissions of IVOCs. These two improvements lead to increased organic carbon concentration in summer but a decreased level in winter. The changes vary by season as a result of differences in volatility (as dictated by temperature and boundary layer height) and reaction rate between winter and summer. Therefore, the missing S/IVOCs and related SOA chemistry in v5.0.2 are key reasons for the OC overprediction and underprediction during cooler and warmer months, respectively.

## 5 Conclusions

In this work, the air quality forecast for the year 2019 predicted by the offline-coupled GFSv15–CMAQv5.0.2 system is comprehensively evaluated. The GFSv15–CMAQv5.0.2 system is found to perform well in predicting surface meteorological variables (temperature, relative humidity, and wind) and O_3_ but has mixed performance for PM_2.5_. Moderate cold biases and wet biases are found in the spring season, especially in March. While the GFSv15–CMAQv5.0.2 system can generally capture the monthly accumulated precipitation compared to remote-sensing and ensemble datasets, temporal distributions of hourly precipitation show less consistency with in situ monitoring data.

MDA8 O_3_ is slightly overpredicted and underpredicted in ozone and the non-O_3_ seasons, respectively. The significant overprediction near the Gulf Coast is associated with the missing halogen chemistry, overestimated emission of precursors, and the poorer performance in meteorological performance, which could be attributed to the missing model representation of the air–sea interaction processes. It compensates for underprediction in the west and Midwest in the O_3_ season for nationwide metrics. A slight underprediction is found during the non-O_3_ season, indicating the impact of cold biases of *T*2 and the overestimated dry deposition to the snow surface. GFSv15–CMAQv5.0.2 has poorer performance in predicting PM_2.5_, compared to the performance for O_3_. Significant overpredictions are found in cooler months, especially in winter. The largest overprediction is shown in the Midwest and the states of Washington and Oregon due mainly to high concentrations of predicted fine fugitive, coarse-mode, and OC compositions. The lacking suppression of snow cover on anthropogenic fugitive dust emission and the non-volatile approach for POA emission contribute a major portion of the overprediction in winter. Meanwhile, the forecasting system may be improved through updating the emissions inventory used (i.e., NEI 2014) to NEI 2016v2 or NEI 2017, which are more representative of the year of 2019 in the next development of next-generation NAQFC.

Categorical evaluation indicates that the GFSv15–CMAQv5.0.2 can capture well the air quality classification of the Moderate category described by the AQI. However, the categorical performance is poorer for PM_2.5_ at the Unhealthy for sensitive groups threshold due mainly to the significant overprediction during the cooler months. Region-specific evaluation further discusses the biases and underlying causes in the 10 U.S. EPA defined regions in CONUS. An update from CMAQv5.0.2 to v5.3.1 is expected to alleviate potential errors in missing sources and mechanisms for SOA formation. The variations of performance between O_3_ and non-O_3_ seasons, as well as during the daytime and nighttime, indicate that further studies need to be conducted to improve boundary layer mixing processes within GFSv15–CMAQv5.0.2. The varied region-specific performance indicates that improvements, such as bias corrections, should be considered individually from region to region in the subsequent development of the next-generation NAQFC.

We have used bias analyses in this work to identify several areas of weakness in the GFSv15–CMAQv5.0.2 system for further improvement and development of next-generation NAQFC. The ability of FV3-based GFS in driving the real-time air quality forecasting is demonstrated. Further studies are still needed to improve the accuracy in meteorological forecast, the emissions, the aerosol chemistry, and the boundary layer mixing for the future GFS–FV3–CMAQ system.

## Supplementary Material

Supplement1

## Figures and Tables

**Table 1.** Configuration of GFSv15–CMAQv5.0.2 system.

Attribute	Model configuration
Forecast period	January–December 2019
Domain	Contiguous US
Resolution	Horizontal: 12 km (442 × 265); vertical: 35 layers
Physical Options	
Shortwave or longwave radiation	The Rapid Radiative Transfer Method for GCMs
Planetary boundary layer (PBL)	Hybrid eddy-diffusivity mass-flux (EDMF) PBL
Land surface	Noah Land Surface Model (LSM)
Microphysics	A more advanced GFDL microphysics scheme
Cumulus	The simplified Arakawa–Schubert (SAS) deep convection
Chemical options	
Photolysis	Inline method (Binkowski et al., 2007)
Gas-phase chemistry	The Carbon Bond mechanism version 5 with active chlorine chemistry and updated toluene mechanism (CB05tucl) (Yarwood et al., 2005; Sarwar etal., 2012)
Aqueous-phase chemistry	AQCHEM (Sarwar et al., 2011)
Aerosol module	AERO6 with non-volatile POA (Carlton et al., 2010; Simon and Bhave, 2012; Appel et al., 2013)

**Table 2.** Performance statistics of meteorological forecasts.

Datasets		CASTNET							METAR						
		
Variable	Period	Mean Obs.	Mean Sim.	MB	RMSE	NMB, %	NME, %	Corr	Mean Obs.	Mean Sim.	MB	RMSE	NMB, %	NME, %	Corr
*T*2, °C	DJF	−0.1	−0.5	−0.4	2.6	−588	−2850	0.96	2.7	2.6	−0.1	2.5	−3.1	69.3	0.97
	MAM	9.9	9.4	−0.5	2.4	−5.2	18.2	0.97	12.3	11.9	−0.4	2.3	−3.0	14.0	0.97
	JJA	21.5	21.4	−0.2	2.4	−0.8	8.6	0.93	23.4	23.1	−0.3	2.3	−1.2	7.5	0.93
	SON	11.5	11.3	−0.2	2.6	−2.0	16.1	0.97	13.8	13.8	0.1	2.3	0.4	12.6	0.98
	Annual	10.9	10.6	−0.3	2.5	−3.0	17.0	0.98	13.2	13.0	−0.2	2.3	−1.3	13.1	0.98

RH2, %	DJF	69.1	71.9	2.8	14.3	4.0	15.1	0.74	74.1	74.4	0.4	13.3	0.5	13.4	0.76
	MAM	62.7	66.1	3.4	14.2	5.4	16.6	0.82	67.4	70.1	2.7	13.8	4.0	15.5	0.81
	JJA	55.0	53.3	−1.7	12.2	−3.2	16.4	0.89	67.0	67.3	0.3	13.1	0.5	14.8	0.84
	SON	59.0	57.6	−1.4	13.0	−2.4	16.1	0.87	68.7	67.0	−1.7	13.2	−2.5	14.5	0.83
	Annual	61.4	62.2	0.8	13.5	1.3	16.0	0.85	68.8	69.3	0.4	13.2	0.8	14.4	0.83

WS10, ms^−1^	DJF	2.5	3.0	0.5	2.0	18.7	56.7	0.59	3.3	3.7	0.4	2.0	10.8	43.5	0.71
	MAM	2.8	3.4	0.6	2.1	22.2	55.6	0.60	3.6	4.0	0.4	2.0	10.3	42.5	0.71
	JJA	2.4	3.0	0.6	1.9	24.5	60.9	0.51	2.8	3.3	0.5	1.9	17.0	52.6	0.62
	SON	2.6	3.1	0.5	2.0	20.4	58.6	0.57	4.0	4.1	0.2	1.8	4.2	33.1	0.69
	Annual	2.6	3.1	0.6	2.0	21.5	57.9	0.57	3.4	3.7	0.4	1.9	10.7	41.8	0.72

WD10, °	DJF	187.2	189.4	2.2	69.4	1.2	26.4	0.81	158.0	164.3	6.4	60.7	4.0	25.5	0.90
	MAM	184.6	186.5	1.9	68.1	1.0	26.1	0.81	159.9	163.6	3.7	60.7	2.3	25.4	0.89
	JJA	186.7	188.8	2.1	73.0	1.1	28.5	0.77	146.8	147.8	1.0	69.9	0.7	33.9	0.86
	SON	181.8	183.9	2.1	71.3	1.1	28.1	0.79	190.9	196.6	5.7	42.1	3.0	14.5	0.92
	Annual	185.0	187.1	2.1	70.5	1.1	27.3	0.80	162.5	166.6	4.1	59.1	2.5	23.9	0.89

Precip, mm h^−1^	DJF	1.0	0.6	−0.4	1.7	−42.5	86.1	0.26	1.3	0.7	−0.6	3.5	−44.4	77.4	0.15
	MAM	1.1	0.6	−0.6	2.0	−51.1	86.3	0.22	1.8	0.7	−1.0	7.5	−58.6	85.6	0.07
	JJA	2.2	0.5	−1.7	4.7	−77.8	93.9	0.11	2.6	0.7	−1.9	7.6	−74.5	91.6	0.04
	SON	1.3	0.6	−0.7	2.4	−54.4	86.2	0.24	1.8	0.8	−1.0	8.8	−56.4	83.8	0.07
	Annual	1.3	0.6	−0.7	2.5	−55.4	87.9	0.18	1.8	0.7	−1.1	7.0	−59.1	85.0	0.07

*T*2: temperature at 2 m; RH2: relative humidity at 2 m; WS10: wind speed at 10 m; WD10: wind direction at 10 m; Precip: precipitation; DJF: winter; MAM: spring; JJA: summer; SON: autumn; MB: mean bias; RMSE: root mean square error; NMB: normalized mean bias; NME: normalized mean error; Corr.: correlation coefficient; Obs.: observation; Sim.: prediction.

**Table 3.** Performance statistics of chemical variables compared to the AIRNow dataset.

	MDA8 O_3_, ppb							24 h average PM_2.5_, μg m^−3^							
	
Period	Mean Obs.	Mean Sim.	MB	RMSE	NMB, %	NME, %	Corr	Period	Mean Obs.	Mean Sim.	MB	RMSE	NMB, %	NME, %	Corr
January	32.1	32.0	−0.1	7.2	−0.4	17.2	0.58	January	8.2	13.8	5.5	11.5	66.9	92.3	0.35
February	36.4	35.5	−0.9	7.8	−2.5	16.7	0.58	February	7.9	12.5	4.6	10.0	58.0	81.5	0.53
March	44.9	40.4	−4.5	8.7	−10.0	15.8	0.56	March	7.8	11.0	3.2	9.2	41.2	69.0	0.40
April	46.4	43.1	−3.3	7.7	−7.1	13.3	0.62	April	6.3	8.0	1.7	6.3	27.9	61.6	0.33
May	44.1	42.7	−1.4	7.8	−3.3	13.9	0.67	May	6.7	6.9	0.2	4.7	3.3	49.3	0.26
June	45.7	43.9	−1.8	10.9	−4.0	18.3	0.59	June	7.1	6.8	−0.3	5.4	−4.2	47.1	0.22
July	44.3	46.6	2.3	9.5	5.2	16.6	0.72	July	8.4	8.5	0.1	11.8	1.0	59.8	0.28
August	43.7	46.9	3.2	9.4	7.3	16.4	0.74	August	7.2	6.9	−0.3	4.0	−4.7	40.2	0.33
September	42.5	45.6	3.1	8.0	7.2	14.4	0.79	September	7.0	7.6	0.6	4.7	8.5	44.2	0.48
October	37.0	40.4	3.4	7.8	9.3	15.8	0.80	October	6.6	9.6	3.0	9.0	44.7	73.2	0.36
November	34.2	35.9	1.8	7.6	5.2	16.5	0.72	November	8.9	13.2	4.2	9.8	47.2	72.1	0.48
December	31.7	33.5	1.8	7.8	5.6	18.6	0.68	December	8.8	13.9	5.1	10.8	57.9	82.5	0.51
O_3_ season	44.1	45.1	1.0	9.2	2.5	16.0	0.69	DJF	8.3	13.4	5.1	10.8	61.0	85.5	0.46
								MAM	6.9	8.6	1.7	7.0	24.8	60.4	0.36
Non-O_3_ season	37.7	37.5	−0.2	7.8	−0.4	16.0	0.72	JJA	7.6	7.4	−0.2	7.8	−2.5	49.5	0.27
								SON	7.5	10.1	2.6	8.1	34.4	63.8	0.46

Annual	40.5	40.9	0.4	8.5	1.0	16.0	0.73	Annual	7.6	9.9	2.3	8.5	30.0	65.2	0.41

MDA8 O_3_: maximum daily average 8 h ozone; 24 h average PM_2.5_: 24 h average PM_2.5_.

## Data Availability

The documentation and source code of CMAQv5.0.2 are available at https://doi.org/10.5281/zenodo. 1079898 (United States Environmental Protection Agency, 2014). The GFS forecast inputs in binary (NEMSIO) format and the coupler used in this study for the GFSv15–CMAQv5.0.2 system are available upon request. The AIRNow data is available for download through the US EPA AirData website (https://www.epa.gov/airdata, US EPA, 2020a). The CASTNET data are available for download from https://www.epa.gov/castnet (US EPA, 2020b). The METAR data are available for download from https://madis.ncep.noaa.gov (NOAA, 2020a). The GPCP data are available through the NOAA website (https://www.ncei.noaa.gov/data/global-precipitation-climatology-project-gpcp-monthly, NOAA 2020b). The CCPA precipitation data are available upon request. The MODIS_MOD04 dataset is available at https://doi.org/10.5067/MODIS/MOD04_L2.006 (Levy and Hsu, 2015). The data processing and analysis scripts are available upon request.
