# Supplementary material for: Evaluation of the offline-coupled GFSv15–FV3–CMAQv5.0.2 in support of the next-generation National Air Quality Forecast Capability over the contiguous United States
Source: Geosci Model Dev. Author manuscript; Available in PMC 2022 Jun 29. (PMC8340608; doi:10.5194/gmd-14-3969-2021)
Supplement: Supplement1 [file NIHMS1724324-supplement-Supplement1.pdf]

Supplement of Geosci. Model Dev., 14, 3969–3993, 2021
https://doi.org/10.5194/gmd-14-3969-2021-supplement
© Author(s) 2021. CC BY 4.0 License.

Geoscientific Model Development
Open Access

Supplement of

Evaluation of the offline-coupled GFSv15–FV3–CMAQv5.0.2 in support of the next-generation National Air Quality Forecast Capability over the contiguous United States

Xiaoyang Chen et al.

Correspondence to: Yang Zhang (ya.zhang@northeastern.edu)

The copyright of individual parts of the supplement might differ from the article licence.

Supplement

Table S1. Datasets used in this study

Database	Parameter	Data frequency	Number of sites / resolution
CASTNET	T2, RH2, WS10, WD10, Precipitation	Hourly	Up to 90 sites
METAR	T2, RH2, WS10, WD10, Precipitation	Hourly	Up to 1900 sites
AirNow	O_3, $PM_{2.5}$	Hourly	Up to 1100 sites
AQS	$PM_{2.5}$ speciation	Daily	Up to 280 sites
CCPA	Precipitation	Hourly	0.125 degree
GPCP	Precipitation	Monthly	2.5 degree
MODIS_MOD04	Aerosol Optical depth	Monthly	1 degree

Table S2. Monthly performance statistics of meteorological variables

Datasets		CASTNET							METAR						
Variable	Period	Mean Obs.	Mean Sim.	MB	RMSE	NMB, %	NME, %	Corr	Mean Obs.	Mean Sim.	MB	RMSE	NMB, %	NME, %	Corr
T2, ºC	Jan	-1.8	-2.2	-0.4	2.5	-23.2	102.1	0.96	1.3	1.1	-0.1	2.4	-11.5	144.5	0.97
	Feb	-0.6	-1.2	-0.6	2.6	-88.8	308.4	0.97	2.5	2.2	-0.2	2.6	-10.2	77.5	0.97
	Mar	3.5	2.7	-0.7	2.4	-20.6	53.2	0.97	6.2	5.8	-0.4	2.3	-6.2	28.2	0.97
	Apr	11.2	10.7	-0.5	2.3	-4.5	16.0	0.96	13.5	13.1	-0.3	2.3	-2.4	12.7	0.96
	May	15.0	14.7	-0.3	2.3	-2.1	11.7	0.96	17.5	17.2	-0.4	2.3	-2.2	9.7	0.96
	Jun	19.8	19.5	-0.3	2.4	-1.7	9.2	0.94	21.9	21.4	-0.5	2.4	-2.1	8.3	0.94
	Jul	22.9	22.9	-0.1	2.5	-0.2	8.2	0.91	24.6	24.4	-0.3	2.3	-1.1	7.1	0.92
	Aug	21.8	21.6	-0.2	2.4	-0.7	8.4	0.93	23.6	23.5	-0.1	2.3	-0.5	7.3	0.93
	Sept	19.5	19.2	-0.3	2.3	-1.7	9.0	0.95	21.7	21.6	-0.1	2.2	-0.6	7.5	0.95
	Oct	11.1	11.0	-0.2	2.7	-1.6	16.7	0.95	13.4	13.5	0.1	2.3	0.6	12.9	0.97
	Nov	4.0	3.8	-0.2	2.8	-4.9	48.5	0.94	6.2	6.5	0.2	2.5	3.8	29.4	0.96
	Dec	2.0	1.8	-0.3	2.8	-12.6	97.6	0.94	4.3	4.4	0.1	2.5	2.4	43.5	0.96
RH2, %	Jan	68.6	70.9	2.3	14.4	3.3	15.3	0.73	72.7	72.3	-0.4	13.1	-0.6	13.6	0.76
	Feb	69.3	74.0	4.6	14.3	6.7	14.8	0.76	74.1	75.6	1.5	14.0	2.0	13.9	0.75
	Mar	63.1	69.1	6.0	15.9	9.6	18.5	0.77	66.6	70.0	3.4	14.7	5.1	16.8	0.77
	Apr	59.9	63.3	3.4	13.3	5.7	16.4	0.85	66.0	68.9	2.9	13.8	4.4	15.9	0.82
	May	65.0	65.6	0.7	13.0	1.0	14.9	0.85	69.5	71.3	1.8	13.0	2.6	14.1	0.82
	Jun	56.4	55.6	-0.8	12.2	-1.4	15.9	0.88	66.4	68.0	1.6	13.7	2.5	15.6	0.82
	Jul	55.0	52.7	-2.3	12.4	-4.2	16.8	0.88	66.6	67.1	0.5	12.9	0.8	14.7	0.84
	Aug	53.7	51.6	-2.0	12.0	-3.8	16.4	0.89	68.0	66.8	-1.1	12.7	-1.7	14.1	0.85
	Sept	58.3	56.0	-2.3	11.7	-3.9	14.7	0.89	68.3	66.9	-1.4	12.3	-2.0	13.6	0.85
	Oct	56.5	54.4	-2.1	12.6	-3.7	16.2	0.89	67.8	65.6	-2.2	13.2	-3.2	14.5	0.85

	Nov	62.4	62.6	0.2	14.5	0.3	17.3	0.82	70.1	68.6	-1.5	14.1	-2.1	15.4	0.77
	Dec	69.3	71.1	1.9	14.3	2.7	15.1	0.75	87.8	88.0	0.2	9.5	0.2	8.0	0.60
WS10, m s^{-1}	Jan	2.6	3.0	0.4	2.0	15.5	55.2	0.61	3.4	3.8	0.3	2.0	9.9	42.5	0.73
	Feb	2.7	3.4	0.6	2.2	22.8	56.9	0.57	3.5	3.8	0.3	2.0	8.2	42.6	0.71
	Mar	2.7	3.2	0.5	1.9	17.0	51.6	0.63	3.6	3.9	0.3	1.9	8.2	41.0	0.72
	Apr	2.9	3.6	0.7	2.2	24.9	57.3	0.58	3.8	4.2	0.4	2.1	11.0	41.3	0.71
	May	2.7	3.3	0.7	2.1	24.7	58.0	0.59	3.4	3.8	0.4	2.0	12.4	45.7	0.66
	Jun	2.6	3.2	0.6	2.1	23.0	60.6	0.51	3.1	3.5	0.5	2.0	14.8	49.3	0.63
	Jul	2.4	3.0	0.6	1.9	25.8	61.5	0.49	2.7	3.2	0.4	1.9	16.2	52.2	0.62
	Aug	2.3	2.8	0.6	1.8	24.8	60.6	0.52	2.6	3.1	0.5	1.9	20.6	57.0	0.59
	Sept	2.6	3.2	0.6	2.0	24.7	58.2	0.59	3.8	4.0	0.2	1.7	5.1	34.3	0.65
	Oct	2.7	3.2	0.5	2.2	20.6	59.7	0.53	4.1	4.3	0.2	1.9	5.1	33.0	0.69
	Nov	2.4	2.8	0.4	2.0	15.3	58.0	0.58	4.0	4.1	0.1	1.8	2.6	32.3	0.70
	Dec	2.4	2.8	0.4	1.9	18.7	58.2	0.57	3.1	3.6	0.4	1.9	14.4	45.8	0.70
Precip, mm hr^{-1}	Jan	1.0	0.6	-0.5	1.6	-43.2	87.1	0.24	1.3	0.8	-0.6	2.9	-44.3	75.9	0.21
	Feb	1.0	0.6	-0.4	1.7	-41.7	86.5	0.25	1.3	0.6	-0.6	4.5	-48.9	78.4	0.11
	Mar	1.1	0.6	-0.5	1.7	-46.0	83.4	0.28	1.6	0.6	-1.0	12.5	-60.5	83.4	0.04
	Apr	1.1	0.5	-0.6	1.9	-54.2	88.4	0.28	1.7	0.8	-0.9	3.5	-51.0	85.2	0.18
	May	1.2	0.5	-0.7	2.3	-55.6	88.8	0.15	2.0	0.7	-1.2	4.9	-63.2	87.3	0.10
	Jun	2.0	0.5	-1.5	4.2	-74.5	93.0	0.09	2.4	0.7	-1.7	5.7	-70.7	89.7	0.07
	Jul	2.2	0.4	-1.8	4.8	-82.4	95.8	0.04	2.8	0.7	-2.1	7.3	-76.1	93.3	0.04
	Aug	2.3	0.5	-1.8	5.2	-77.2	93.1	0.17	2.7	0.6	-2.1	9.7	-77.3	92.1	0.03
	Sept	1.3	0.5	-0.8	2.4	-60.5	86.8	0.18	2.3	0.7	-1.6	13.7	-70.2	89.9	0.03
	Oct	1.5	0.7	-0.8	2.7	-52.4	88.0	0.25	1.9	0.9	-0.9	6.7	-49.9	82.8	0.10
	Nov	1.1	0.5	-0.5	1.9	-49.8	82.7	0.28	1.3	0.6	-0.7	6.1	-50.6	77.7	0.07
	Dec	1.0	0.6	-0.4	1.5	-44.2	83.7	0.32	1.4	0.8	-0.6	2.7	-40.4	77.7	0.18

Table S3. Summary of forecasting skills of air quality forecasting systems

Reference	Region	Model System	Period	Pollutant	Performance
Moran et al., 2018	Canada/North America	GEM-MACH	2010	O_3	NMB=1.4%, R=0.76
				$PM_{2.5}$	NMB=-0.6%, R=0.58
Russell et al., 2019	Canada	GEM-MACH	Aug-Sept 2013	O_3	MB=5.7 to 10.9 ppb, RMSE=9.7 to 16.0 ppb, Corr=0.50 to 0.74
				$PM_{2.5}$	MB=3.2 to 5.5 μg m^{-3}, RMSE=5.7 to 8.8 μg m^{-3}, Corr=0.20 to 0.47
Struzewska et al., 2016	Poland	GEM-AQ	Nov 2011 to Sep 2013	O_3	MB=12.8 to 25.6 μg m^{-3}, RMSE=24.6 to 28.7 μg m^{-3}, Corr=0.48 to 0.62
				$PM_{2.5}$	MB=-9.6 to -1.86 μg m^{-3}, RMSE=24.8 to 34.1 μg m^{-3}, Corr=0.48 to 0.58
D'Allura et al., 2018	Italy	WRF/RAMS-FARM	2015	O_3	FAR=36.1%, POD= 71.2%, threshold=83 μg m^{-3}
				PM_{10}	FAR=20.0%, POD= 27.3%, threshold=33 μg m^{-3}
Podrascanin, 2019	Serbia	WRF/Chem	August 2016	O_3	MB=1.6 to 9.3 μg m^{-3}, NMB=3.0 to 17.2%, Corr=0.45 to 0.50
				PM_{10}	MB=-15.2 to -14.3 μg m^{-3}, NMB=-74.0 to -56.1%, Corr=-0.01 to 0.18
Stortini et al., 2020	Italy	CHIMERE	October 2019	O_3	MB=11.0 to 16.9 μg m^{-3}, RMSE=19.3 to 28.0 μg m^{-3}, Corr=0.63 to 0.78
				PM_{10}	MB=-8.2 to -4.9 μg m^{-3}, RMSE=11.4 to 13.0 ug m-3, Corr=0.72 to 0.76, FAR=43-44%, POD= 6-22%

Lyu et al., 2017	China	WRF-CMAQ	2014-2016	$PM_{2.5}$	NME=49%, RMSE=32.2 ug m-3, R2=0.46
Zhou et al., 2017	China	WRF/Chem	2014-2015	MDA8 O_3	MB=18.9 ppb, NMB=77%, RMSE=27.9 ppb, Corr=0.63
				$PM_{2.5}$	MB=-12.0 μg m^{-3},NMB=-9%, RMSE=35.8 μg m^{-3}, Corr=0.67
Peng et al., 2018	China	WRF/Chem	6 to 16 October 2014	O_3	MB=-31.0 μg m^{-3}, RMSE=50.8 μg m^{-3}, Corr=0.46
				$PM_{2.5}$	MB=-34.1 μg m^{-3}, RMSE=92.1 μg m^{-3}, Corr=0.74
Ha et al., 2020	Korea	WRF/Chem	May 2016	$PM_{2.5}$	MAE=12.8, FAR=59.0%, Overall_Accuracy=59.7%, High_Pollution_Accuracy=35.6%
Kang et al., 2010	CONUS	NAM-CMAQ	2008	MDA8 O_3	HR=~47% for cat 2, ~31% for cat 3, FAR=~68% at cat 3
				$PM_{2.5}$	HR=~46% for cat 2, ~21% for cat3, FAR=~91% at cat 3
Zhang et al., 2016	Southeastern US	WRF/Chem-MADRID	2012-2014 (May–September)	MDA8 O_3	NMB= 0.0 to 17.0 %, NME= 22.0 to 27.0 %, Corr= 0.5
				$PM_{2.5}$	NMB= -4.0 to 15.0 %, NME= 36.0 to 40.0 %, Corr= 0.3 to 0.4
			2012-2014 (December-February)	MDA8 O_3	NMB= -17.7 to -9.1 %, NME= 19.6 to 24.6 %, Corr= 0.0 to 0.2
				$PM_{2.5}$	NMB= 0.8 to 8.3 %, NME= 42.6 to 47.2 %, Corr= 0.3 to 0.4
Lee et al., 2017	CONUS	NAM-CMAQ	May and Jul 2014	$PM_{2.5}$	MB=-2.7 to -1.6 μg m^{-3},NME=-35 to -20%, RMSE=4.5 to 8.8 μg m^{-3}, Corr=0.22 to 0.33
			Jan 2015	$PM_{2.5}$	MB=1.3 to 3.7 μg m^{-3}, NME=13 to 38%, RMSE=6.5 to 12.6 μg m^{-3}, Corr=0.37 to 0.38
This work	CONUS	FV3GFS-CMAQ	2019	MDA8 O_3	MB=0.4 ppb, NMB=1.0%, Corr=0.73. FAR=41.4 % and HR=45.8% at cat 2.
				$PM_{2.5}$	MB=2.3 μg m^{-3}, NMB=30.0%, Corr=0.41. FAR=70.3% and HR=57.6% for cat 2.

FAR: False Alarm Ratio; POD: Probability of Detection; HR: Hit Rate.

Figure S1. Site locations within CONUS of (a) CASTNET and (b) METAR. (c) Ten regions within CONUS for regional analysis

Figure S2. Spatial distribution of MBs for forecasted seasonal mean T2 by GFSv15-CMAQv5.0.2 against observations from METAR

Figure S3. Monthly accumulated precipitation for four seasons by (left column) the GFSv15-CMAQv5.0.2 prediction, (middle column) the CCPA observation, and (right column) the GPCP observation

Figure S4. Time series of observed and predicted hourly precipitation at two CASTNET sites at site GRS420 in

Tennessee during (a) Jan, (b) July, (c) Aug, and (d) Nov.

Figure S5. Spatial distribution of annual area emissions

Figure S6. Time series of daily maximum 8-hr O_3 in 10 regions within CONUS

Figure S7. Diurnal O_3 in: (a) O_3 season for regions 1 to 5; (b) Non-O_3 season for regions 1 to 5; (c) O_3 season for regions 6 to 10; (d) non-O_3 season for regions 6 to 10

Figure S8. Time series of 24-h avg $PM_{2.5}$ in 10 regions within CONUS for regions 6 to 10; (d) Non-O_3 season for region 6 to 10. Solid curves are observed values and dash curves are predicted values

Figure S9. Annual performance for 10 regions in predicting meteorological variables of temperature at 2-m (T2), relative humidity at 2-m (RH2), precipitation, and wind speed at 10-m (WS10)

Figure S10. Difference of VOC emissions from pt_oilgas sector in 2016 NEI comparing to the emissions used in

Figure S11. (a) Monthly variation of domain-wide surface emissions, and (b) diurnal emissions of fine mode PM

(a) (b)

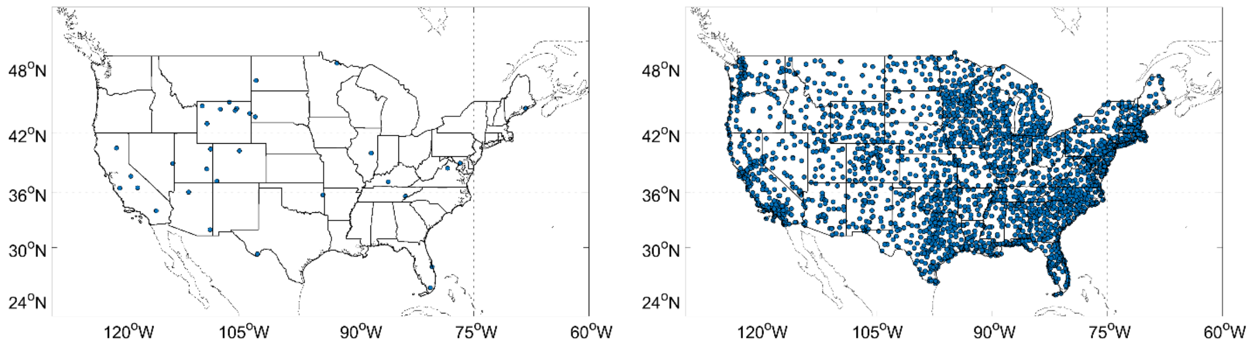

(c)

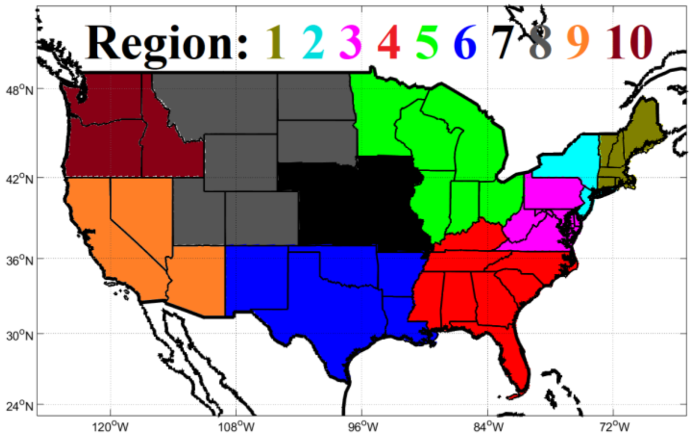

Figure S1. Site locations within CONUS of (a) CASTNET and (b) METAR. (c) Ten regions within CONUS for regional analysis: (Region 1) Connecticut, Maine, Massachusetts, New Hampshire, Rhode Island, and Vermont; (Region 2) New Jersey, and New York; (Region 3) Delaware, Washington D.C, Maryland, Pennsylvania, Virginia, and West Virginia; (Region 4) Alabama, Florida, Georgia, Kentucky, Mississippi, North Carolina, South Carolina, and Tennessee; (Region 5) Illinois, Indiana, Michigan, Minnesota, Ohio, and Wisconsin; (Region 6) Arkansas, Louisiana, New Mexico, Oklahoma, and Texas; (Region 7) Iowa, Kansas, Missouri, and Nebraska; (Region 8) Colorado, Montana, North Dakota, South Dakota, Utah, and Wyoming; (Region 9) Arizona, California, and Nevada; and (Region 10) Idaho, Oregon, and Washington

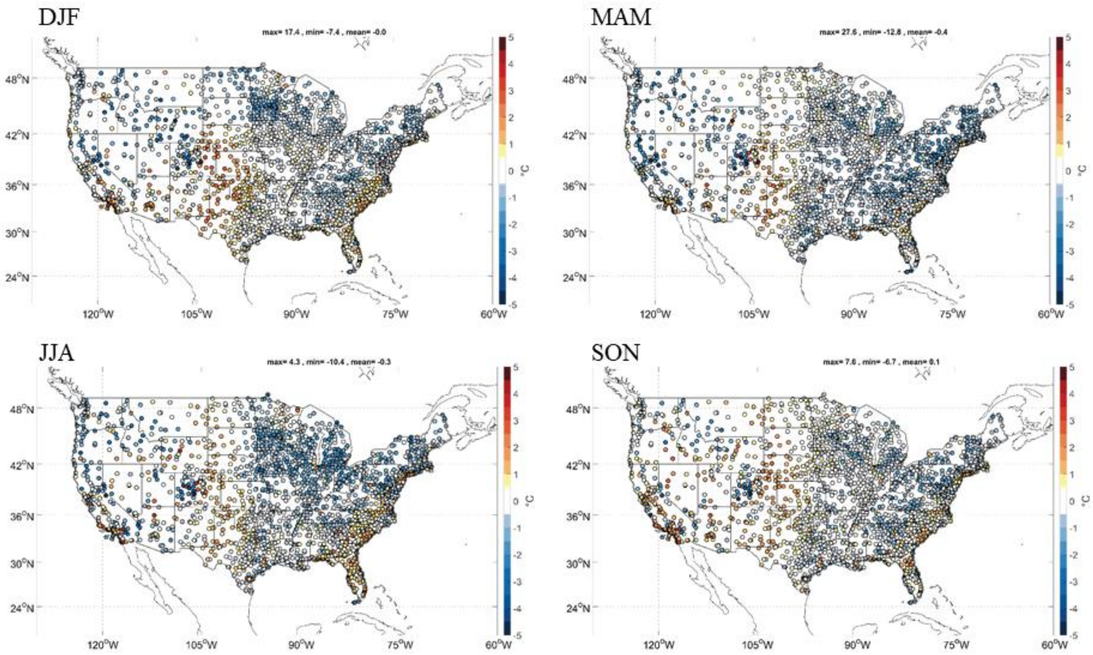

Figure S2. Spatial distribution of MBs for forecasted seasonal mean T2 by GFSv15-CMAQv5.0.2 against observations from METAR

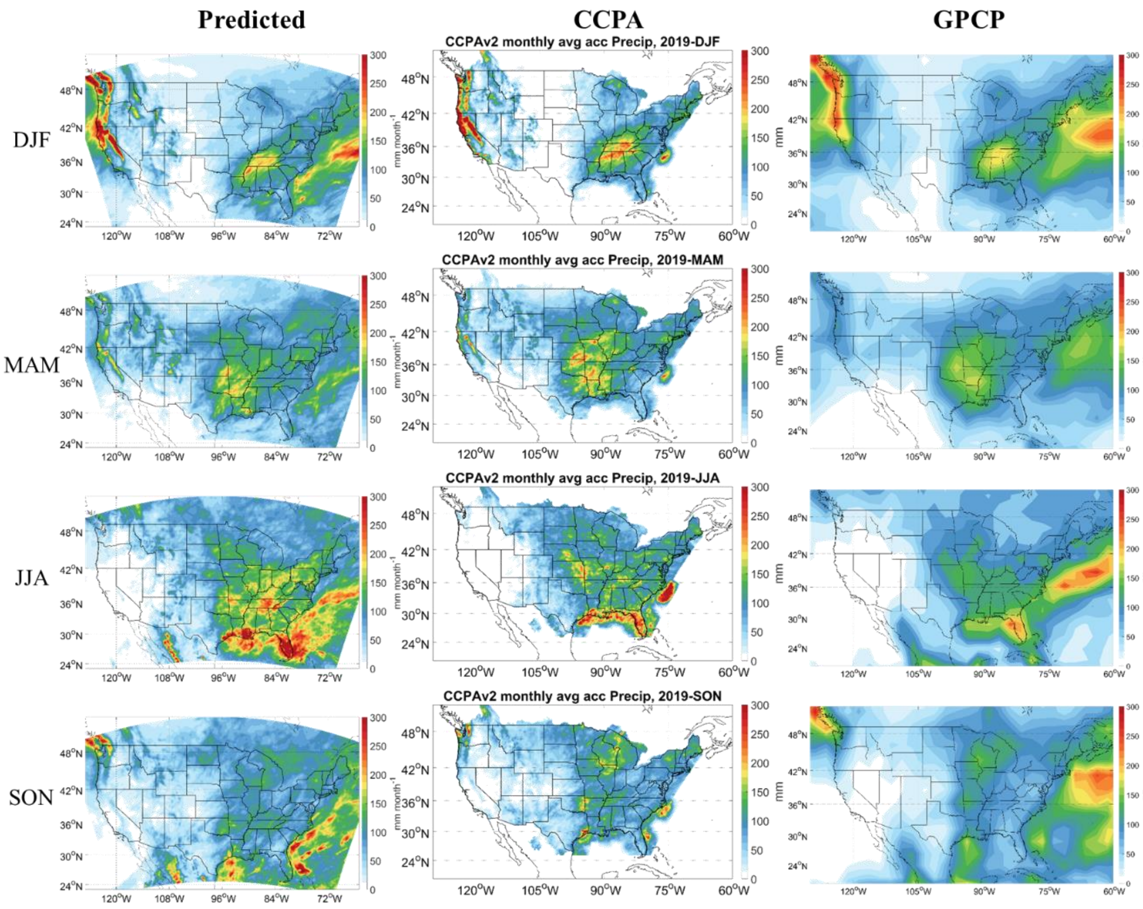

Figure S3. Monthly accumulated precipitation for four seasons by (left column) the GFSv15-CMAQv5.0.2 prediction, (middle column) the CCPA observation, and (right column) the GPCP observation

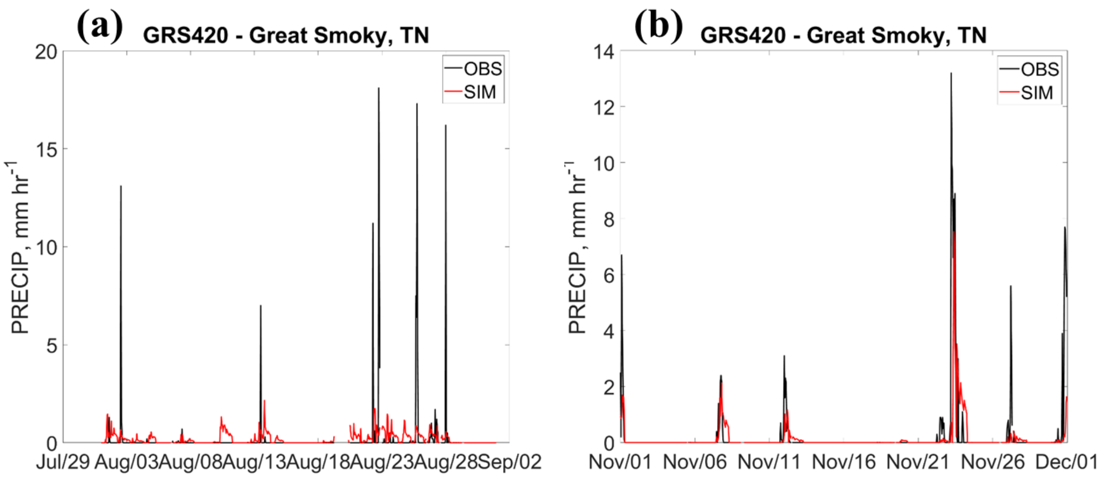

Figure S4. Time series of observed and predicted hourly precipitation at two CASTNET sites at site GRS420 in

Tennessee during (a) Jan, (b) July, (c) Aug, and (d) Nov.

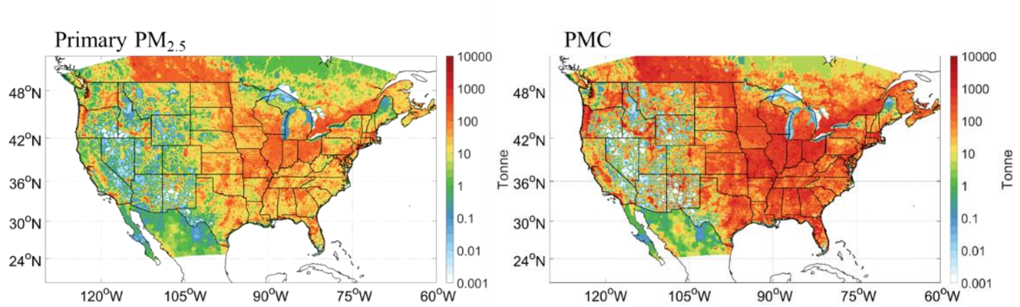

Figure S5. Spatial distribution of annual area emissions in anthropogenic sectors for primary $PM_{2.5}$, and coarse mode PM (PMC)

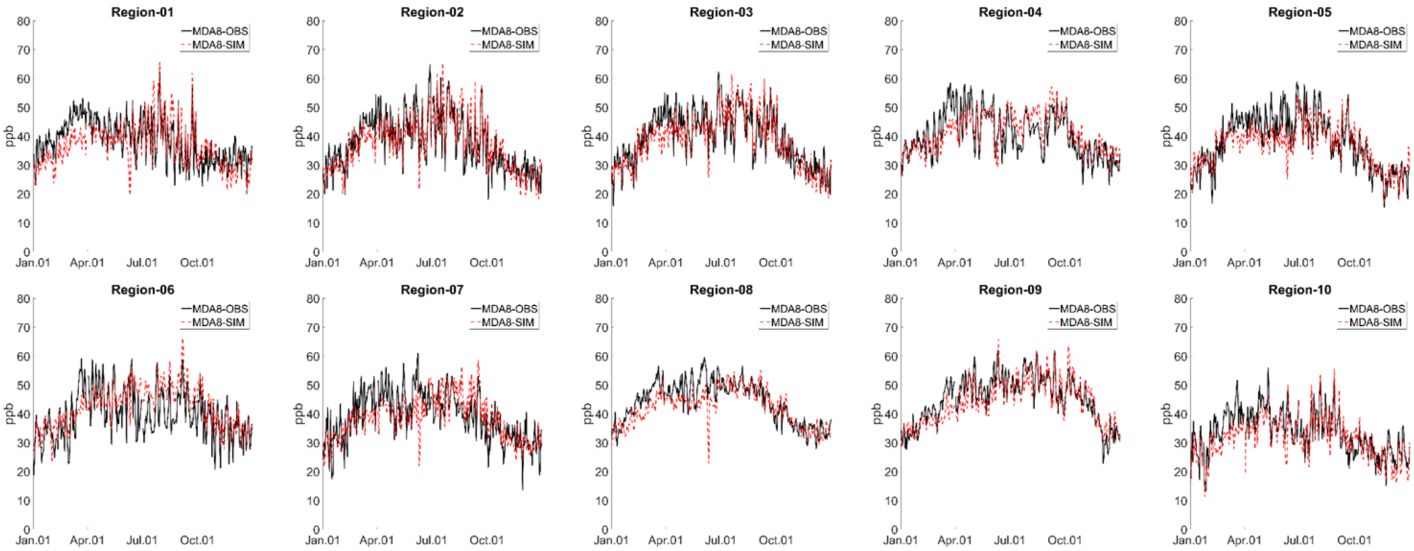

Figure S6. Time series of daily maximum 8-hr O_3 in 10 regions within CONUS

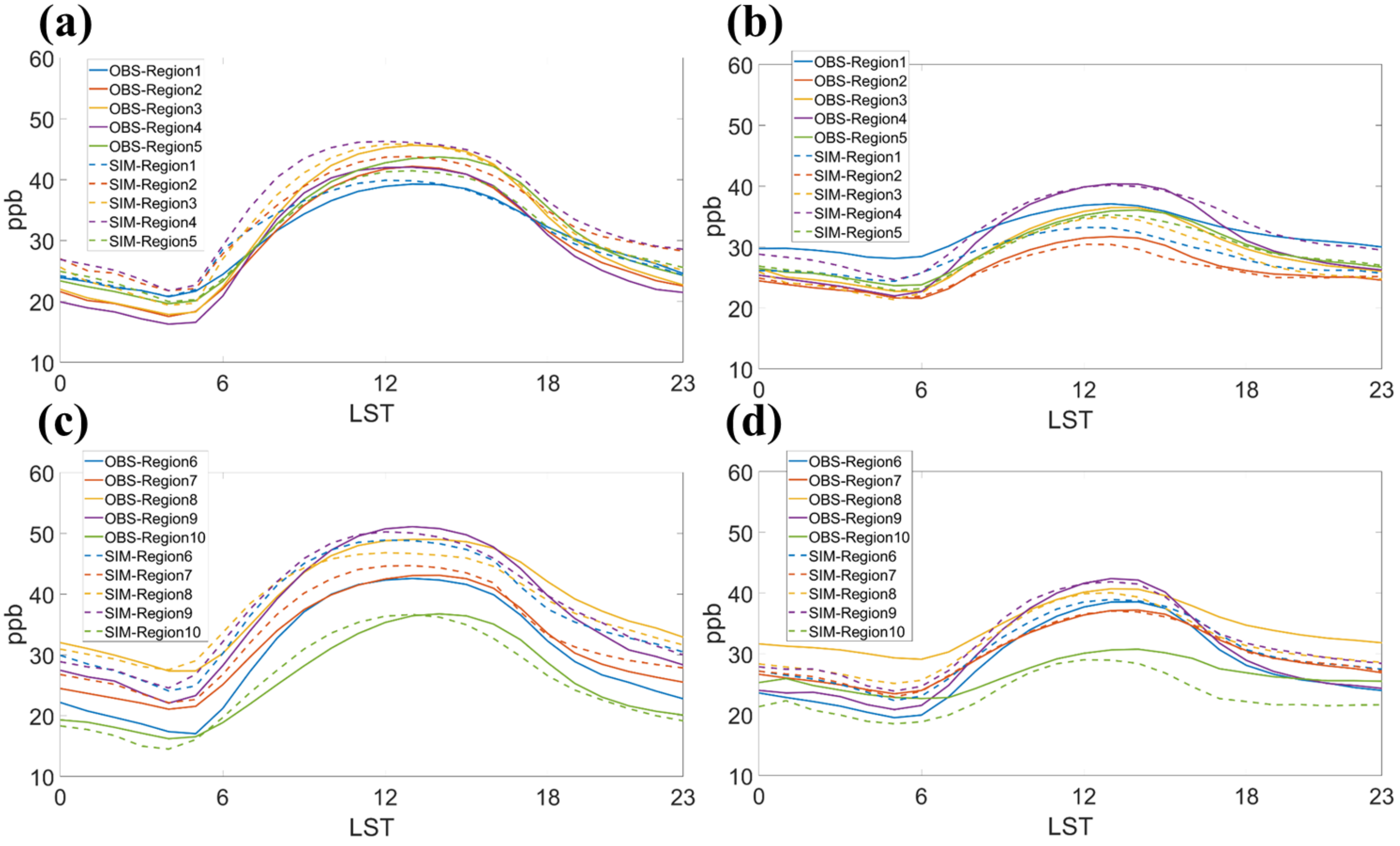

Figure S7. Diurnal O_3 in: (a) O_3 season for regions 1 to 5; (b) Non-O_3 season for regions 1 to 5; (c) O_3 season for regions 6 to 10; (d) non-O_3 season for regions 6 to 10

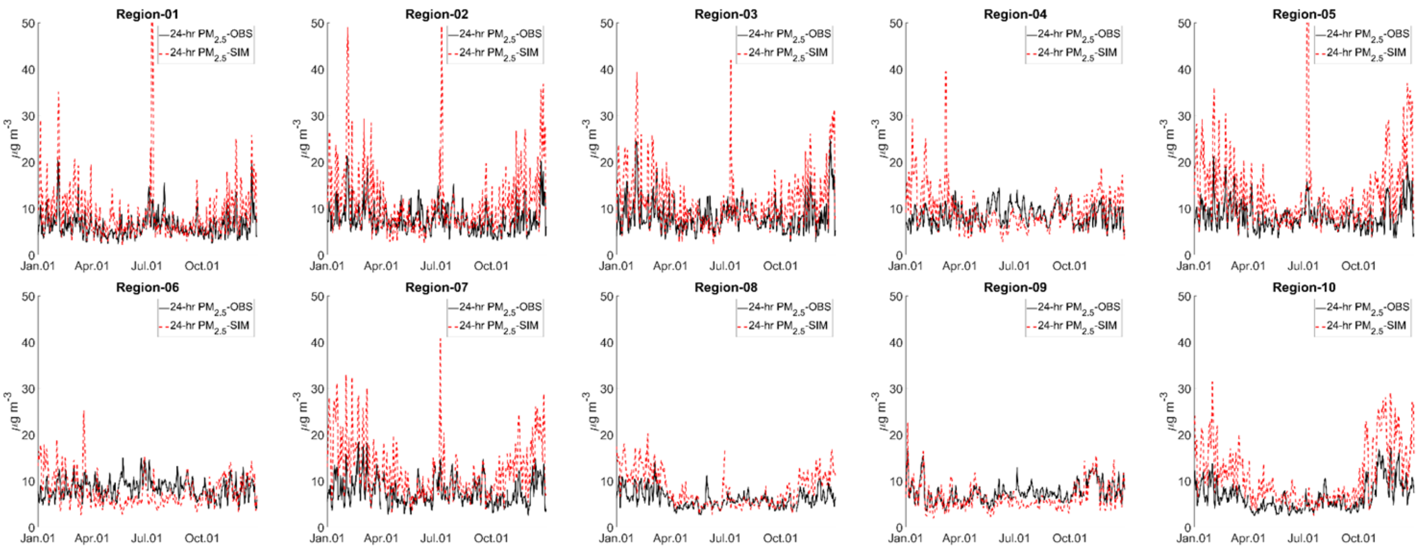

Figure S8. Time series of 24-h avg $PM_{2.5}$ in 10 regions within CONUS for regions 6 to 10; (d) Non-O_3 season

for region 6 to 10. Solid curves are observed values and dash curves are predicted values

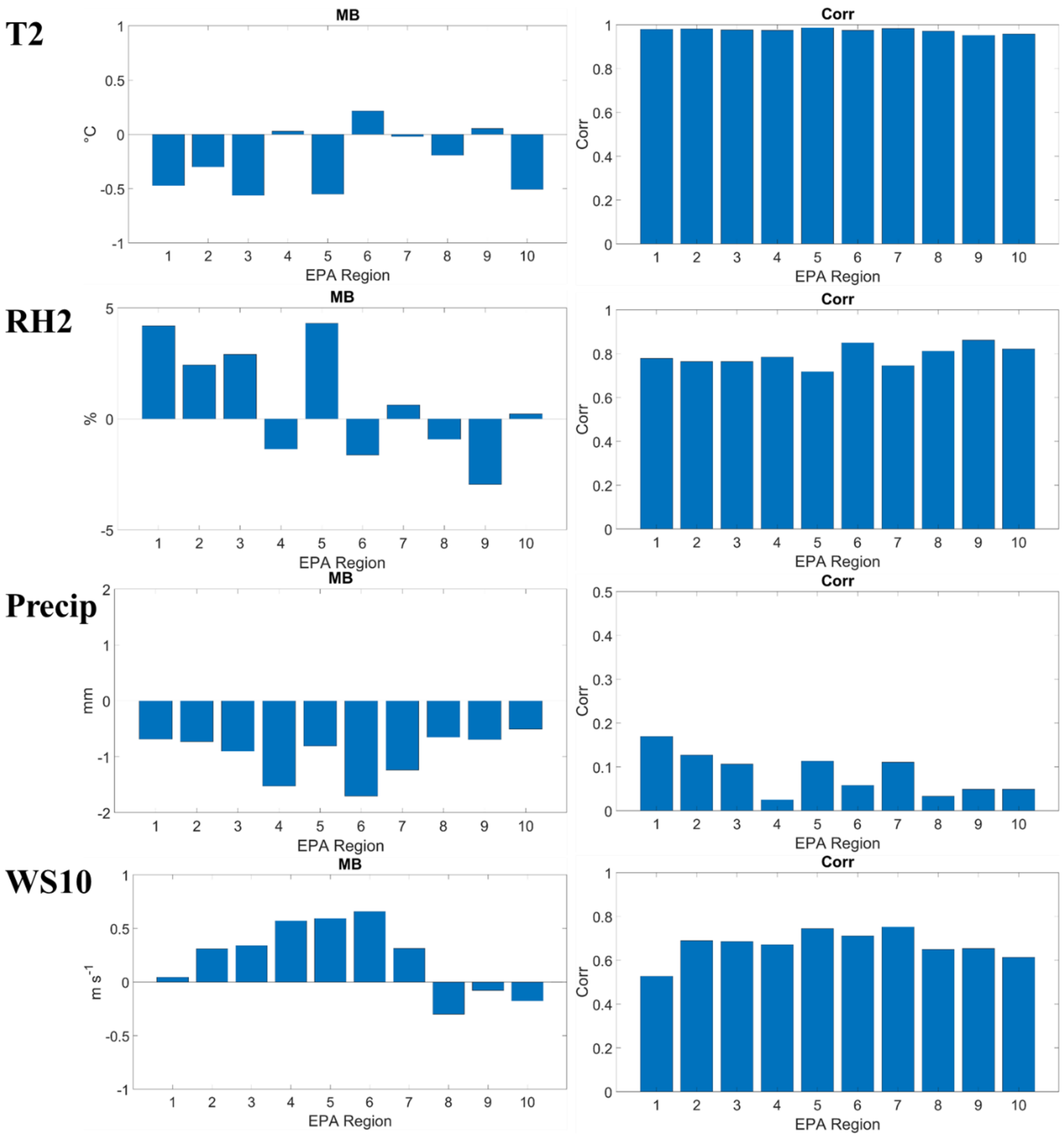

Figure S9. Annual performance for 10 regions in predicting meteorological variables of temperature at 2-m (T2), relative humidity at 2-m (RH2), precipitation, and wind speed at 10-m (WS10)

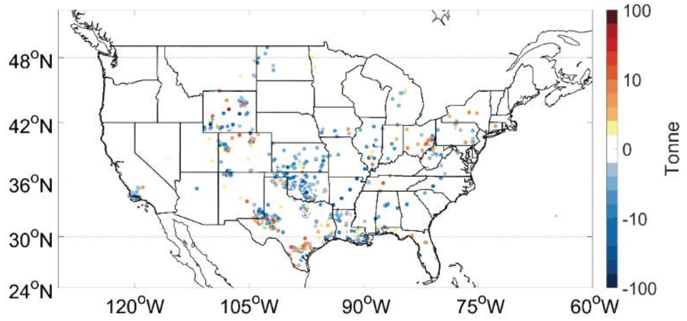

Figure S10. Difference of VOC emissions from pt_oilgas sector in 2016 NEI comparing to the emissions used in this study

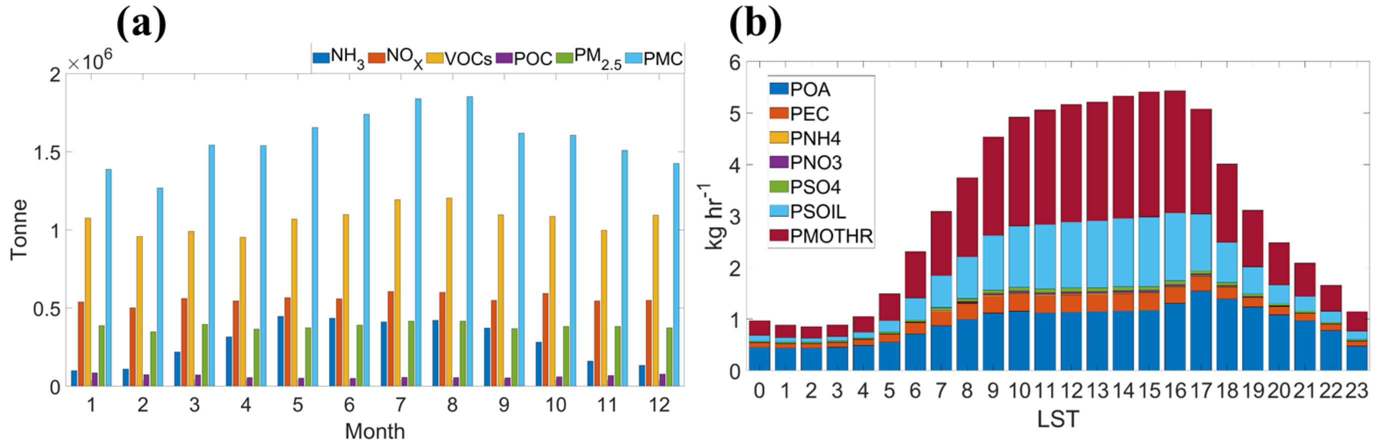

Figure S11. (a) Monthly variation of domain-wide surface emissions, and (b) diurnal emissions of fine mode PM

References

Appel, K. W., Pouliot, G. A., Simon, H., Sarwar, G., Pye, H. O. T., Napelenok, S. L., Akhtar, F. and Roselle, S. J.: Evaluation of dust and trace metal estimates from the Community Multiscale Air Quality (CMAQ) model version 5.0, Geosci. Model Dev., 6(4), 883–899, doi:10.5194/gmd-6-883-2013, 2013.

D'Allura, A., Costa, M. P., & Silibello, C. (2018). Qualearia: European and national scale air quality forecast system performance evaluation. International Journal of Environment and Pollution, 64(1–3), 110–124. https://doi.org/10.1504/IJEP.2018.099152

Ha, S., Liu, Z., Sun, W., Lee, Y., & Chang, L. (2020). Improving air quality forecasting with the assimilation of GOCI aerosol optical depth (AOD) retrievals during the KORUS-AQ period. Atmospheric Chemistry and Physics, 20(10), 6015–6036. https://doi.org/10.5194/acp-20-6015-2020

Kang, D., Mathur, R., & Trivikrama Rao, S. (2010). Real-time bias-adjusted O3 and PM2.5 air quality index forecasts and their performance evaluations over the continental United States. Atmospheric Environment, 44(18), 2203–2212. https://doi.org/10.1016/j.atmosenv.2010.03.017

Lee, P., McQueen, J., Stajner, I., Huang, J., Pan, L., Tong, D., et al. (2017). NAQFC Developmental Forecast Guidance for Fine Particulate Matter (PM 2.5) . Weather and Forecasting, 32(1), 343–360. https://doi.org/10.1175/waf-d-15-0163.1

Lyu, B., Zhang, Y., & Hu, Y. (2017). Improving PM2.5 Air Quality Model Forecasts in China Using a Bias-Correction Framework. Atmosphere, 8(12), 147. https://doi.org/10.3390/atmos8080147

Moran, M. D., Lupu, A., Zhang, J., Savic-Jovcic, V., & Gravel, S. (2018). A comprehensive performance evaluation of the next generation of the canadian operational regional air quality deterministic prediction system. In Springer Proceedings in Complexity (pp. 75–81). Springer. https://doi.org/10.1007/978-3-319-57645-9_12

Oliveri Conti, G., Heibati, B., Kloog, I., Fiore, M., & Ferrante, M. (2017). A review of AirQ Models and their applications for forecasting the air pollution health outcomes. Environmental Science and Pollution Research, 24(7), 6426–6445. https://doi.org/10.1007/s11356-016-8180-1

Peng, Z., Lei, L., Liu, Z., Sun, J., Ding, A., Ban, J., et al. (2018). The impact of multi-species surface chemical observation assimilation on air quality forecasts in China. Atmospheric Chemistry and Physics, 18(23), 17387–17404. https://doi.org/10.5194/acp-18-17387-2018

Podrascanin, Z. (2019). Setting-up a Real-Time Air Quality Forecasting system for Serbia: a WRF-Chem feasibility study with different horizontal resolutions and emission inventories. Environmental Science and Pollution Research, 26(17), 17066–17079. https://doi.org/10.1007/s11356-019-05140-y

Russell, M., Hakami, A., Makar, P. A., Akingunola, A., Zhang, J., Moran, M. D., & Zheng, Q. (2019). An evaluation of the efficacy of very high resolution air-quality modelling over the Athabasca oil sands region, Alberta, Canada. Atmospheric Chemistry and Physics, 19(7), 4393–4417. https://doi.org/10.5194/acp-19-4393-2019

Sarwar, G., Fahey, K., Napelenok, S., Roselle, S. and Mathur, R.: Examining the impact of CMAQ model updates on aerosol sulfate predictions, the 10th Annual CMAS Models-3 User's Conference, Chapel Hill, NC, October 2011, 2011.

Sarwar, G., Simon, H., Bhave, P. and Yarwood, G.: Examining the impact of heterogeneous nitryl chloride production on air quality across the United States. Atmospheric Chemistry and Physics, 12(14), 6455–6473. https://doi.org/10.5194/acp-12-6455-2012, 2012.

Simon, H., and Bhave, P. V.: Simulating the degree of oxidation in atmospheric organic particles. Environmental Science and Technology, 46(1), 331–339. https://doi.org/10.1021/es202361w, 2012.

Spiridonov, V., Jakimovski, B., Spiridonova, I., & Pereira, G. (2019). Development of air quality forecasting system in Macedonia, based on WRF-Chem model. Air Quality, Atmosphere and Health, 12(7), 825–836. https://doi.org/10.1007/s11869-019-00698-5

Stortini, M., Arvani, B., & Deserti, M. (2020). Operational forecast and daily assessment of the air quality in Italy: A copernicus-CAMS downstream service. Atmosphere, 11(5), 447. https://doi.org/10.3390/ATMOS11050447

Struzewska, J., Kaminski, J. W., & Jefimow, M. (2016). Application of model output statistics to the GEM-AQ high resolution air quality forecast. Atmospheric Research, 181, 186–199. https://doi.org/10.1016/j.atmosres.2016.06.012

Taylor, K. E.: Summarizing multiple aspects of model performance in a single diagram. Journal of Geophysical Research: Atmospheres, 106(D7), 7183–7192. https://doi.org/10.1029/2000JD900719, 2001.

Yarwood, G., Rao, S., Yocke, M. and Whitten, G.: Updates to the Carbon Bond Chemical Mechanism: CB05.

Final Report to the US EPA, RT-0400675. Yocke and Company, Novato, CA, 2005.

Zhang, Y., Hong, C., Yahya, K., Li, Q., Zhang, Q., & He, K. (2016). Comprehensive evaluation of multi-year real-time air quality forecasting using an online-coupled meteorology-chemistry model over southeastern United States. Atmospheric Environment, 138, 162–182. https://doi.org/10.1016/j.atmosenv.2016.05.006

Zhou, G., Xu, J., Xie, Y., Chang, L., Gao, W., Gu, Y., & Zhou, J. (2017). Numerical air quality forecasting over eastern China: An operational application of WRF-Chem. Atmospheric Environment, 153, 94–108. https://doi.org/10.1016/j.atmosenv.2017.01.020
